# c-Myc shuttled by tumour-derived extracellular vesicles promotes lung bronchial cell proliferation through miR-19b and miR-92a

**DOI:** 10.1038/s41419-019-2003-5

**Published:** 2019-10-07

**Authors:** Cristina Borzi, Linda Calzolari, Anna M. Ferretti, Laura Caleca, Ugo Pastorino, Gabriella Sozzi, Orazio Fortunato

**Affiliations:** 1 Tumor Genomics Unit, Department of Research, Fondazione IRCCS Istituto Nazionale dei Tumori, 20133 Milan, Italy; 2 Lab. Di nanotecnologie, Istituto di scienze e tecnologie Molecolari CNR, 20138 Milan, Italy; 3 Unit of Molecular Bases of Genetic Risk and Genetic Testing, Fondazione IRCCS Istituto Nazionale dei Tumori, 20133 Milan, Italy; 4 Thoracic Surgery Unit, Fondazione IRCCS Istituto Nazionale dei Tumori, 20133 Milan, Italy

**Keywords:** Non-small-cell lung cancer, miRNAs

## Abstract

Lung cancer causes approximately one fifth of all cancer deaths. Tumour cells actively communicate with the surrounding microenvironment to support malignant progression. Extracellular vesicles (EVs) play a pivotal role in intercellular communication and modulate recipient cells by delivering their contents, including proteins and nucleic acids such as microRNAs (miRNAs). We isolated EVs from the conditioned medium (CM) of human lung cancer cell lines and plasma of lung cancer patients and cancer-free smokers using an ultracentrifugation method. A significant increase in bronchial HBEC-KRAS^V12high^ cell proliferation, confirmed by cell cycle analysis, was observed after treatment with cancer-derived EVs. Lung cancer-derived EVs induced transcription of the pri-miR-92a gene, resulting in the overexpression of mature miR-19b and miR-92a in recipient bronchial cells. Modulation of these two miRNAs using miRNA mimics or inhibitors confirmed their ability to promote proliferation. In silico analysis and experimental validation showed that miR-19b and miR-92a impaired the TGF-beta (TGFB) pathway and identified TGFBRI and TGFBRII as target genes involved in EV-mediated bronchial cell proliferation. Interestingly, the oncoprotein c-Myc, a well-known miR-17-92 cluster activator, was detected only in the EVs derived from lung cancer patients and cell lines and was able to modulate the proliferation of HBEC-KRAS^V12high^ recipient cells. These data support the role of c-Myc shuttling in lung cancer-derived EVs in inducing the upregulation of onco-miR-19b and miR-92a expression with concomitant impairment of the TGFB signalling pathway in recipient cells.

## Introduction

Lung cancer is one of the most common and malignant types of cancer worldwide^1,2^. In 2018, it was estimated that lung cancer accounted for 14% of all new diagnoses and ~25% of all cancer deaths^3^. The high mortality is due to the absence of symptoms in early stages^4^ and the lack of effective therapeutic interventions, despite improvements achieved in recent years with immunotherapy^2,5^. Therefore, to improve survival, there is still a clinical need to investigate the molecular mechanisms underlying lung cancer development and progression. Lung tumourigenesis is strongly regulated by the complex interplay between tumour and stromal cells, including fibroblasts^6^, endothelial cells^7^ and immune cells^5,8^. Interactions among cells in the tumour microenvironment are mediated by several mechanisms: cell–cell contact (receptor-mediated interactions and gap junctions) and paracrine signals (growth factors, cytokines and chemokines) as well as by extracellular vesicles (EVs)^9,10^.

EVs are spherical, bilayered, membranous vesicles generated by all cell types in mammalian organisms. EVs are generally recognised as exosomes (30–150 nm), microvesicles (0.1–1 µm) and apoptotic bodies (0.8–5 µm). EVs are constitutively released by different cell types to mediate cell-to-cell communication both in normal and pathological states^11^. EVs contain a heterogeneous composition of biomolecules, including lipids, proteins and nucleic acids such as DNA and RNA (mRNAs, long non-coding RNAs, microRNAs (miRNAs))^11–13^. Cells can use EVs to shuttle biomolecules to neighbouring or distant cells and influence recipient cell functionality^11,14^. Recent evidence shows that EVs, in particular exosomes, are closely related to lung carcinogenesis^15^. Tumour-derived exosomes play a crucial role in the growth and progression of lung cancer by modulating tumour angiogenesis^16^ and epithelial-to-mesenchymal transition (EMT)^17^. In addition, Lobb RJ et al. demonstrated that exosomes derived from oncogenically transformed bronchial epithelial cells transferred chemoresistance to recipient cells through exosomal ZEB1 mRNA transfer^18^.

MiRNAs represent a class of small non-coding RNAs that act as master regulators of gene expression^19^. In cancer, they can act as tumour suppressors or oncogenes^20^. We previously generated a miRNA risk classifier based on the reciprocal ratios of 24 plasma-derived-miRNAs associated with lung cancer development and prognosis^21,22^. We also demonstrated the functional roles of specific miRNAs composing the signature (miR-486 and miR-660) in lung tumourigenesis^23,24^, supporting their central roles in the modulation of cancer-associated pathways, such as the p53. An interesting work showed that miR-21 and miR-29a contained in lung tumour-derived exosomes were able to bind Toll-like receptors (TLRs) expressed by immune cells, leading to a TLR-mediated inflammatory response that supported lung tumour growth and metastasis^25^. It has been shown that exosomal miR-23a, which is overexpressed in hypoxic lung cancer, enhances angiogenesis and vascular permeability through its targets prolyl hydroxylase (PHD) and tight junction protein-1^26^. The same authors identified miR-103a contained in hypoxic lung cancer-derived exosomes as a mediator of M2-phenotype polarisation in macrophages through a PTEN-dependent mechanism^27^.

To date, the functional roles of miRNAs associated with EVs in lung cancer development and progression are largely unknown. The present study aimed to characterise EVs derived from lung cancer cells and patients and elucidate their pro-tumourigenic roles in the lung epithelium, focusing on the functional interactions between EVs and miRNAs. Lung cancer is a multistep process resulting in specific oncogene and/or tumour suppressor gene alterations in epithelial cells due to a prolonged smoke exposure. To this purpose, we utilised non-tumorigenic Bronchial Epithelial HBEC-KRAS^V12high^ as recipient cells. These cells are oncogenically-modified HBECs^28^ that show a certain degree of ‘plasticity’ versus a partial malignant transformation. These features make HBECs-KRAS^V12high^ an appropriate model to investigate the effects of tumour-derived EVs in neighbouring cells and their contribution to lung carcinogenesis.

## Results

### Isolation and characterisation of EVs from lung cancer cell lines

**Fig. 1:**
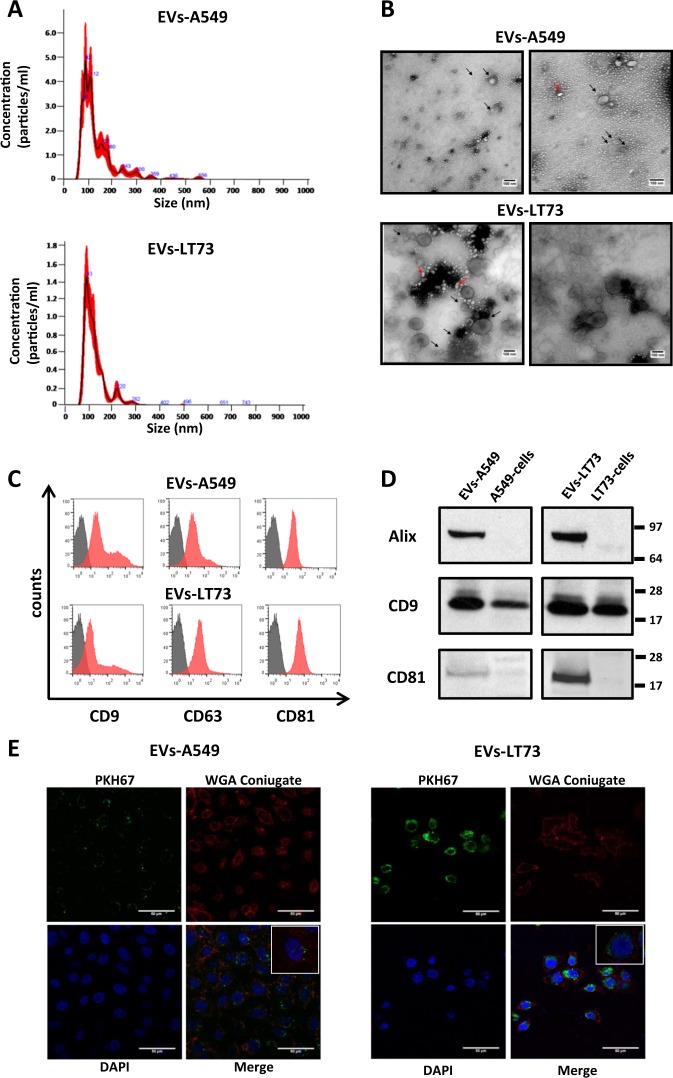
Characterisation of EVs isolated from lung cancer cell lines. **a** Size distribution and concentration of A549 cell- and LT73 cell-derived EVs obtained using the NanoSight Instrument. **b** Representative images of EV pellets observed by TEM. Black arrows indicate EVs, while red arrows represent artefacts. **c**, **d** Analysis of the exosome-enriched proteins CD9, CD63, CD81 and Alix by FACS (**c**, representative images, *n* = 3) and western blotting (**d**). Cellular lysates were used as a control for western blot analysis. **e** Representative confocal laser scanning microscopy images of PKH67-labelled EV uptake by 633-WGA-labelled HBEC-KRAS^V12high^ recipient cells after 24 h. Green = PKH67-labelled EVs, red = WGA-conjugated recipient cells and blue = nuclei

EVs from the lung cancer cell lines A549 and LT73 and HBEC-KRAS^V12high^ were isolated from conditioned medium (CM) and characterised. Size distribution analysis using NanoSight identified a major peak between 50 and 200 nm, with modes of 89 and 96 nm for A549- and LT73-derived EVs (EVs-A549 and EVs-LT73), respectively (Fig. 1a and Table 1). A similar size distribution was observed for EVs derived from HBEC-KRAS^V12high^ cells, with a diameter’s mode about 100 nm (Supplementary Fig. 1a and Table 1). Notably, we observed a greater amount of EVs, approximately four times higher, being released by tumour cells than by HBEC-KRAS^V12high^ cells, as measured by their total protein content (Supplementary Fig. 1b and Table 1).

**Table 1:** Size and concentration of EVs isolated from different lung cell lines

	EVs-A549	EVs-LT73	EVs-HBEC-KRAS^V12high^
Mean size (nm)	143.9 ± 8.7	130.3 ± 6.3	121.9 ± 6.3
Mode size (nm)	89.3 ± 7.3	96.2 ± 4.5	99.6 ± 7.2
Particles/ml	3.6 × 10^10^ ± 2.5 × 10^9^	8.9 × 10^10^ ± 3.9 × 10^9^	9.0 × 10^9^ ± 9.6 × 10^8^

NanoSight analysis was performed using 5 µg of EVs from each cell type.

The results obtained by NanoSight were confirmed by transmission electron microscopy (TEM) analysis. The vesicles showed a spherical shape with a relatively wide size distribution (Fig. 1b, Supplementary Fig. 1c, d). EVs-A549 had a mean diameter (*d*_m_) of 56.7 nm, whereas a larger diameter, *d*_m_ = 69.8 nm, was detected for EVs-LT73. Instead, HBEC-KRAS^V12high^ derived EVs showed the smallest average diameter (*d*_m_ = 38.2) (Supplementary Fig. 1d).

FACS and WB analysis revealed high levels of the exosome-enriched proteins CD9, CD63, CD81 and Alix (Fig. 1c, d, Supplementary Fig. 1e). To explore the pro-tumourigenic potential of lung cancer-derived EVs, we evaluated the in vitro interaction between tumoural EVs and HBEC-KRAS^V12high^, mimicking a possible in vivo interplay between a tumour and the surrounding epithelial component. To this end, we treated HBEC-KRAS^V12high^ cells with PKH67-labelled EVs. After 24 h of culture, confocal laser scanning microscopy images showed the uptake of EVs-A549 and EVs-LT73 by recipient cells (Fig. 1e). FACS analysis confirmed the presence of EVs inside cells at different time points (24, 48 and 72 h) (Supplementary Fig. 2).

### Lung cancer-derived EVs promote bronchial epithelial cell proliferation through miR-19b and miR-92a

**Fig. 2:**
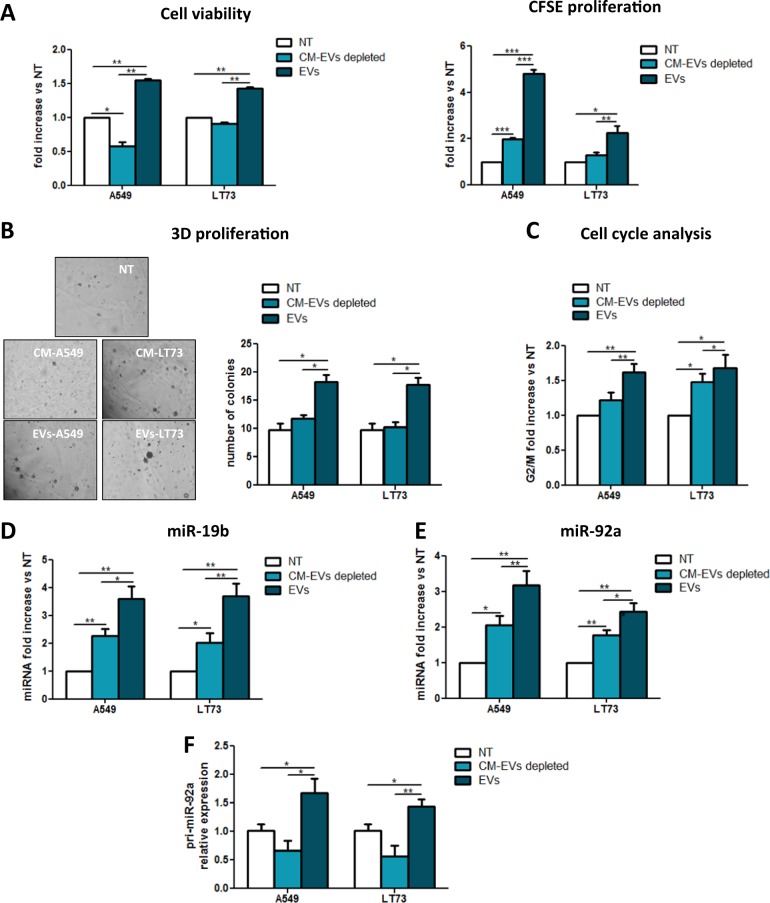
Lung cancer-derived EVs induced epithelial cell proliferation through miR-19b and miR-92a. **a** Evaluation of HBEC-KRAS^V12high^ cell viability (left; Trypan blue cell count, *n* = 3) and proliferation (right; CFSE analysis, *n* = 3) after 72 h of treatment with A549 cell- or LT73 cell-derived EVs or the corresponding EV-depleted CM. Untreated cells (NT) were used as a negative control. **b** 3D proliferation assay using VitroGel. Images show the colony formation ability of HBEC-KRAS^V12high^ cells treated as mentioned above. **c** Graphs show cells in the G2/M phase after EV treatment (*n* = 5). **d**, **e** Relative miR-19b (**d**) and miR-92a (**e**) expression levels in HBEC-KRAS^V12high^ cells after 24 h of the indicated treatment compared to untreated cells (*n* = 5). **f** pri-miR-92a expression levels in untreated, EV-depleted CM- and EV-treated HBEC-KRAS^V12high^ cells. B2m was used as a housekeeping control (*n* = 5). Data are expressed as the mean ± SEM. *p < 0.05, **p < 0.01

To explore the interaction between tumoural EVs and HBEC-KRAS^V12high^, we treated HBEC-KRAS^V12high^ cells with EVs (15 µg) and the corresponding CM-EVs depleted. Untreated cells were used as an additional control. Compared to EV-depleted CM treated and untreated cells, EV-treated HBEC-KRAS^V12high^ cells showed a significant increase in proliferation at 72 h (Fig. 2a). This result was confirmed by carboxyfluorescein succinimidyl ester (CFSE) analysis (Fig. 2a, Supplementary Fig. 3). Moreover, we tested the capability of tumoural EVs to support 3D cell culture. Treatment with EVs-A549 and EVs-LT73 resulted in enhanced 3D proliferation (defined as a cluster of at least 10 cells), as compared to control cells over time (Fig. 2b). In addition, cell cycle analysis revealed a significant increase of cells in the G2/M phase in EV-treated cells compared to controls (Fig. 2c).

To characterise the pro-tumourigenic effects of EVs, we evaluated migration and EMT process in HBEC-KRAS^V12high^ cells after EV treatment. A wound healing assay revealed no difference in wound closure time between the EVs-A549- or EVs-LT73-treated cells and the corresponding controls (Supplementary Fig. 4a). Gene expression analysis did not show significant modulation of EMT-related gene levels (Supplementary Fig. 4b), indicating no alterations in either migratory ability or epithelial phenotype occurred. These results agree with published data, which showed that these cells need to grow in serum-supplemented medium to undergo EMT^28^.

To investigate the involvement of EV-miRNAs in the enhancement of HBEC-KRAS^V12high^ proliferation, we analysed miRNA expression in recipient cells, focusing on 24 miRNAs that we previously implicated in the pathogenesis and aggressiveness of lung tumours^21^. Among the 24 miRNAs, only miR-19b and miR-92a exhibited significant upregulation in recipient cells treated with EVs derived from both cell lines compared to control (Fig. 2d, e, Supplementary Fig. 5). To understand whether EV-shuttled miR-19b and miR-92a could be directly transferred to recipient cells, we evaluated the expression of pri-miR-92a (a precursor of both miRNAs) in treated cells. We observed a statistically significant increase in pri-miR-92a expression in the EV-treated cells as shown in Fig. 2f. Since pri-miR-92a was not detected inside EVs-A549 and EVs-LT73 (data not shown), these results suggest endogenous transcription of pri-miR-92a and, consequently, of the two miRNAs. To exclude miRNAs transfer, we isolated EVs from A549 where miR-19b and miR-92a were silenced by LNA inhibitors, obtaining EVs devoided of the two miRNA (EVs-A549(LNA)) (about 95 and 90% of reduction for miR-19b and miR-92a, respectively) (Supplementary Fig. 6a). HBEC-KRAS^V12high^ exposed to EVs-A549(LNA) showed pro-tumorigenic phenotype as observed for WT-EVs: increase in cell growth and G2/M cell cycle phase (Supplementary Fig. 6b). Upregulation of the two miRNAs was observed in recipient cells upon EVs-A549(LNA) exposure, excluding an EVs-mediated miRNAs transfer (Supplementary Fig. 6c).

**Fig. 3:**
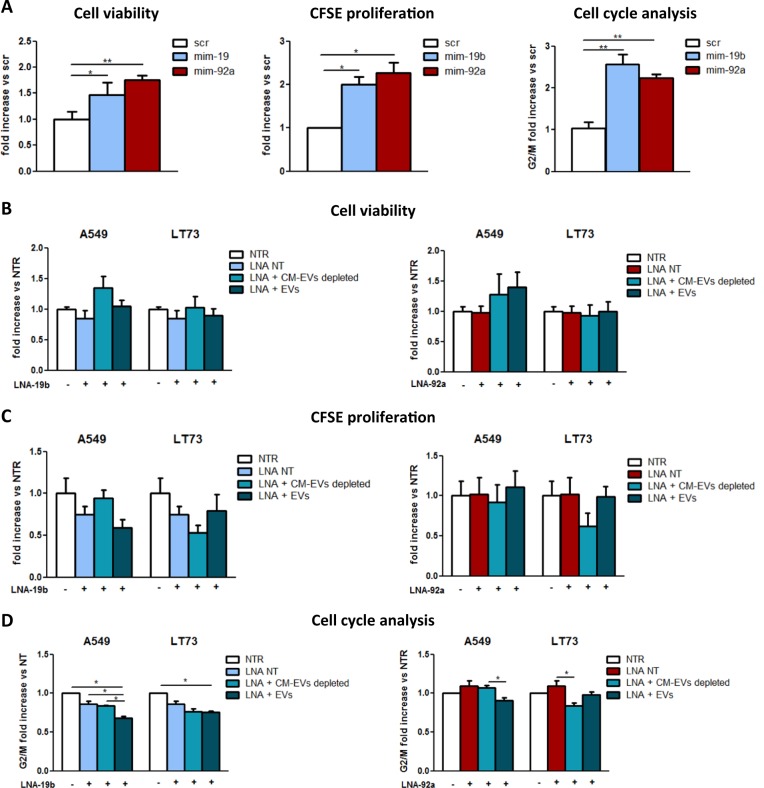
MiR-19b and miR-92a overexpression in HBEC-KRAS^V12high^ cells increased proliferation. **a** Effects of miR-19b and miR-92a overexpression on HBEC-KRAS^V12high^ cell viability (left; Trypan blue cell count, *n* = 5), proliferation (middle; CFSE analysis, *n* = 5) and cell cycle distribution (right; *n* = 5) at 72 h compared to the effects of scramble (scr). **b–d** Inhibition of EV-mediated effects on cell viability (**b**; Trypan blue cell count, *n* = 3), proliferation (**c**; CFSE analysis, *n* = 3) and cell cycle distribution (**d**; *n* = 3) produced by miR-19b (left) and miR-92a (right) inhibition in HBEC-KRAS^V12high^ cells. Data are expressed as the mean ± SEM. *p < 0.05, **p < 0.01, ***p < 0.001

The roles of miR-19b and miR-92a on cell proliferation were also investigated using miRNA mimics (5 nM) in HBEC-KRAS^V12high^ cells, and 20 and 28% increases in cell numbers following miR-19b (mim-19b) or miR-92a (mim-92a) overexpression, respectively, compared to scramble (scr) were observed (*p* < 0.01; Fig. 3a, left). This result was confirmed by CFSE analysis (Fig. 3a, middle) and cell cycle analysis (Fig. 3a, right), where an increase in the proportion of cells in the G2/M phase was detected. miR-19b and miR-92a levels following miRNAs mimics treatment are shown in Supplementary Fig. 7a. Of note, overexpression of the two miRNAs was comparable to those induced by EVs exposure (Supplementary Fig. 7b).

To confirm the crucial roles of these two miRNAs in the aforementioned phenotypic modulation, we performed miR-19b and miR-92a inhibition in recipient cells using LNA inhibitors and then evaluated HBEC-KRAS^V12high^ cell proliferation after EV treatment (Fig. 3b–d, Supplementary Fig. 8). Compared to no treatment, treatment with LNA-19b or LNA-92a caused 90 and 95% reductions in miR-19b and miR-92a expression levels, respectively. Treatment with EVs or EV-depleted CM did not enhance miRNA expression in LNA-treated cells (Supplementary Fig. 8). Accordingly, LNA treatment reversed the advantages conferred by EVs in cell viability (Fig. 3b) and CFSE analyses (Fig. 3c). In fact, inhibition of the miRNAs reduced cell viability and differences in the cell cycle were no longer detected (Fig. 3b–d).

To verify the specificity of the functional effects observed after tumour cell-derived EV exposure, we repeated the experiment using EVs derived from non-tumourigenic HBEC-KRAS^V12high^ cells (EVs- HBEC-KRAS^V12high^). Notably, the proliferation of the recipient cells was not affected by exposure to EVs- HBEC-KRAS^V12high^ (Supplementary Fig. 9a–c).

Overall, these results demonstrated the ability of lung cancer EVs to induce the upregulation of miR-19b and miR-92a expression and increase the proliferation of recipient epithelial cells.

### TGFBRs are involved in EV-mediated epithelial cell proliferation

**Fig. 4:**
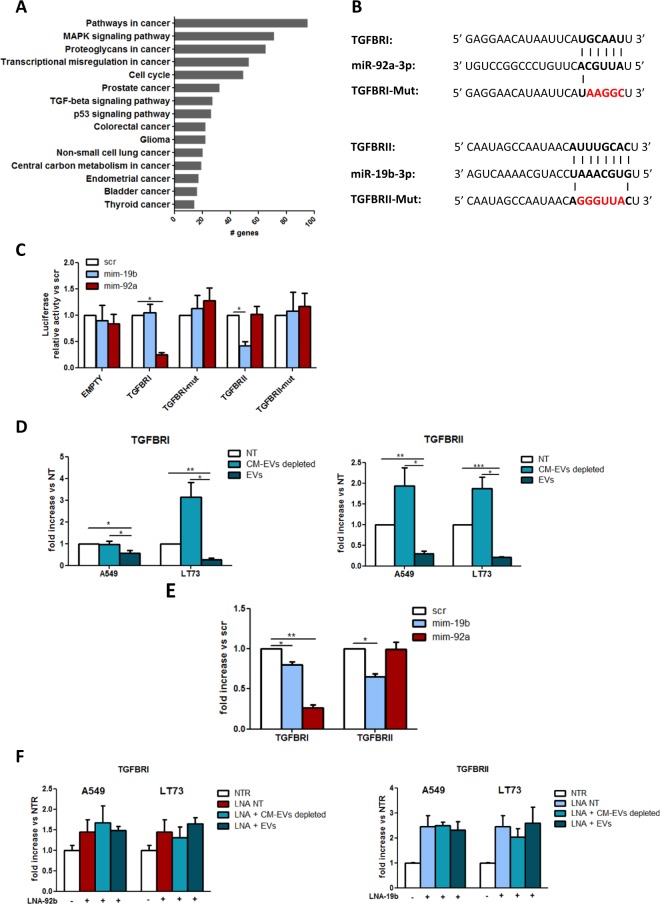
TGFBRs were involved in EV-mediated proliferation. **a** Gene enrichment analysis of experimentally validated miR-19b and miR-92a target genes. Only cancer-related pathways are shown in the graph. **b** miR-92a and miR-19b seed sequence alignments with the 3′ UTRs of TGFBRI and TGFBRII, respectively. Both wild-type and mutated sequences of TGFBRs 3′UTR are shown (mutated sequences are indicated in red). **c** Bar graph showing relative luciferase activity, compared to control, for wild-type and mutated TGFBRI- and TGFBRII-3′UTR reporters. WT and EMPTY control plasmids were transfected into HEK293 cells alone or with mim-19b, mim-92a or scr-control. Reporter activity was measured after 48 h and normalised to Renilla luciferase activity (*n* = 3). **d** Modulation of TGFBRI and TGFBRII protein expression in HBEC-KRAS^V12high^ cells after 72 h of treatment with EVs or EV-depleted CM (*n* = 3). **e** Modulation of TGFBRI and TGFBRII protein expression following miR-19b and miR-92a overexpression (*n* = 3). **f** Effects of miR-92a (left) and miR-19b (right) inhibition on TGFBRI and TGFBRII protein expression, respectively, after the indicated treatments (*n* = 3). Data are expressed as the mean ± SEM. *p < 0.05, **p < 0.01, ***p < 0.001

In silico analyses of experimentally validated miR-19b and miR-92a targets were performed to understand the molecular mechanisms underlying HBEC-KRAS^V12high^ cell proliferation. KEGG pathway analysis revealed associations of miR-19b and miR-92a target genes with cancer pathways, including NSCLC-related pathways, cell cycle-related pathways and TGF-beta (TGFB) signalling, as illustrated in Fig. 4a. Several studies have reported downregulation of TGFB signalling after miR-17–92 overexpression in different types of cancer, including lung cancer^29–31^. Therefore, we focused on the genes involved in TGFB signalling, and target prediction revealed TGFBRI and TGFBRII as potential miR-92a and miR-19b targets, respectively (Fig. 4b). To confirm that TGFBRs are direct targets of miR-19b and miR-92a, we performed a luciferase reporter assay and observed downmodulation of luciferase activity when HEK-293 cells were co-transfected with miRNA mimics (75 and 58% of reduction for TGFBRI-mim-92a and TGFBRII-mim19b, respectively) (Fig. 4c). Target specificity was validated using a 3′UTR EMPTY and mutated 3′UTR-TGFBRs vectors, as depicted in Fig. 4b, where no changes in luciferase activity were detected (Fig. 4c). Therefore, TGFBRI and TGFBRII were experimentally validated as miR-92a and miR-19b targets, respectively, in HBEC-KRAS^V12high^ cells.

Using flow cytometry analysis, we found that the expression of TGFBRI and TGFBRII was downregulated in HBEC-KRAS^V12high^ cells treated with cancer-derived EVs compared to untreated cells (50 and 61% reductions in TGFBRI expression and 44 and 79% reductions in TGFBRII expression for EVs-A549 and EVs-LT73, respectively, p < 0.05) and CM control-treated cells (44 and 73% reductions in TGFBRI expression and 69 and 79% reductions in TGFBRII expression for EVs-A549 and EVs-LT73, respectively, *p* < 0.05) (Fig. 4d and Supplementary Figs. 10a–12). A similar result was observed also using EVs depleted of miR-19b and miR-92a (Supplementary Fig. 6d).

We also observed downmodulation of the respective targets, both at the mRNA (Supplementary Fig. 10b) and protein levels (Fig. 4e, Supplementary Figs. 11 and 12), after miR-19b and miR-92a overexpression. Compared to no treatment, inhibition of miR-19b or miR-92a in HBECs increased the protein level of TGFBRII or TGFBRI, respectively (Fig. 4f). Notably, LNA-92a (Fig. 4f, left) and LNA-19b (Fig. 4f, right) prevented TGFBR downmodulation in EV-treated cells. TGFBRI and TGFBRII expression levels in recipient cells were not affected by EVs-HBEC-KRAS^V12high^ exposure. This is in agreement with the unchanged pri-miR-92a expression level (Supplementary Fig. 9d, e). An impairment of TGFB pathway after EVs exposure was observed through the modulation of downstream targets such as CDC25A, E2F-1, p15, p21, and p57, as already described^32–35^ (Supplementary Fig. 10c).

These data indicate that modulation by miR-19b and miR-92a impairs the TGFBR pathway in bronchial epithelial cells, contributing to the increase in the proliferative rate induced by lung cancer-derived EVs.

### c-Myc shuttled by tumour-derived EVs promotes malignant transformation of epithelial recipient cells

**Fig. 5:**
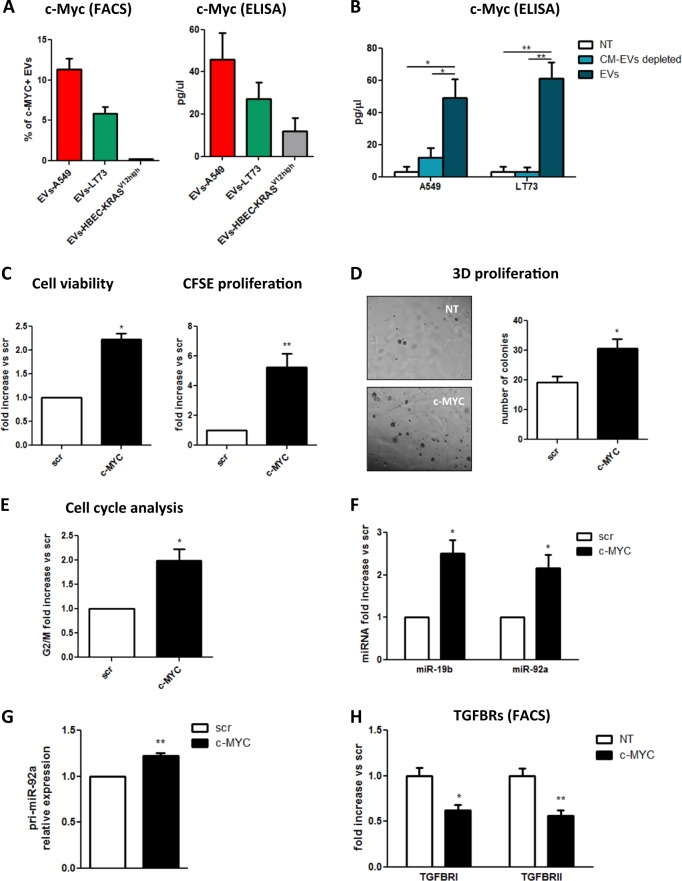
c-Myc shuttled by tumour-derived EVs induced malignant transformation in recipient epithelial cells. **a** c-Myc protein quantification in EVs. Left: percentage of EVs-A549 (red bar), EV-LT73 (green bar) and EVs-HBEC-KRAS^V12high^ (grey bar) positive for c-Myc expression (FACS analysis, *n* = 3); right: absolute quantification (pg/µl) of c-Myc carried by the different EVs (ELISA, *n* = 3). **b** Quantification of c-Myc in HBEC-KRAS^V12high^ after the indicated treatment (ELISA, *n* = 3). **c**–**f** Graphs show cell viability (**c**, left; *n* = 3), CFSE (**c**, right; *n* = 3), 3D proliferation (**d**, *n* = 3) G2/M phase cells (**e**; *n* = 3) and TGFBRI and TGFBRII expression level (**f**, *n* = 3) upon transient transfection of c-Myc plasmid. Data are expressed as mean ± SEM. *p < 0.05, **p < 0.01

To better define the molecular mechanism linking EV treatment to epithelial cell proliferation, we searched for the presence of a transcriptional activator of pri-miR-92a in the EV cargo. The oncoprotein c-Myc is a well-known miR-17–92 cluster activator^36^. Therefore, we tested EVs-A549 and EVs-LT73 for the presence of c-Myc by flow cytometry, and we observed that both samples were positive, showing ~10 and 5% c-Myc^+^-EVs among the EVs-A549 and EVs-LT73, respectively (Fig. 5a, left and Supplementary Fig. 13a).

EVs-A549(LNA) displayed same levels of c-Myc expression compared to A549 wild-type EVs (Supplementary Figs. 6e and 13a). C-Myc protein was detected by WB in A549 and LT73 cells and at lower levels in HBEC-KRAS^V12high^ (Supplementary Fig. 13b). Moreover, c-Myc intracellular expression level and EVs cargo were quantified by enzyme-linked immunosorbent assay (ELISA) (c -Myc: about 1.1 and 1.4 ng/μl for A549 and LT73 cellular lysates, Supplementary Fig. 13c, and 58 and 30 pg/µl for EVs-A549 and EVs-LT73 respectively, Fig. 5a, right), showing that about 4 and 2% of cellular c-Myc was released within EVs by A549 and LT73, respectively. In addition, EVs-HBEC-KRAS^V12high^ had lower amount of c-Myc compared to tumour EVs (Fig. 5a, Supplementary Fig. 13a). To prove c-Myc-shuttling, we measured c-Myc level in HBEC-KRAS^V12high^ after EVs exposure observing a greater increase of c-Myc in both EVs-A549 and EVs-LT73 treated cells compared to controls (Fig. 5b). We detected modulation of miRNAs known to be regulated by c-Myc^36^ such as miR-9, miR-17, miR-18a, miR-34a, let-7b (Supplementary Fig. 13d). These data suggest an EVs-mediated transfer of c-Myc from lung tumour to epithelial cells.

To prove that EV-shuttled c-Myc was responsible for the phenotypic changes observed, we transiently transfected c-Myc plasmid into HBEC-KRAS^V12high^ cells. A significant increase in cell proliferation was observed by both Trypan blue and CFSE analysis (Fig. 5c, Supplementary Fig. 14), an increase in the number of colonies (Fig. 5d), and an enhancement in G2/M phase was noted (Fig. 5e). Overexpression of c-Myc induced pri-miR-92a transcription followed by miR-19b and miR-92a upregulation (Fig. 5f–g). We detected 50% reductions in TGFBRI- and TGFBRII-positive cell numbers in c-Myc-overexpressing cells compared to control (Fig. 5h).

These results suggest a mechanistic explanation for the ability of lung cancer-derived EVs to induce a pro-tumourigenic feature in HBEC-KRAS^V12high^ cells.

### Lung cancer patient-derived EVs enhance bronchial epithelial cells proliferation through EVs shuttled c-Myc –TGFB pathway interplay

**Fig. 6:**
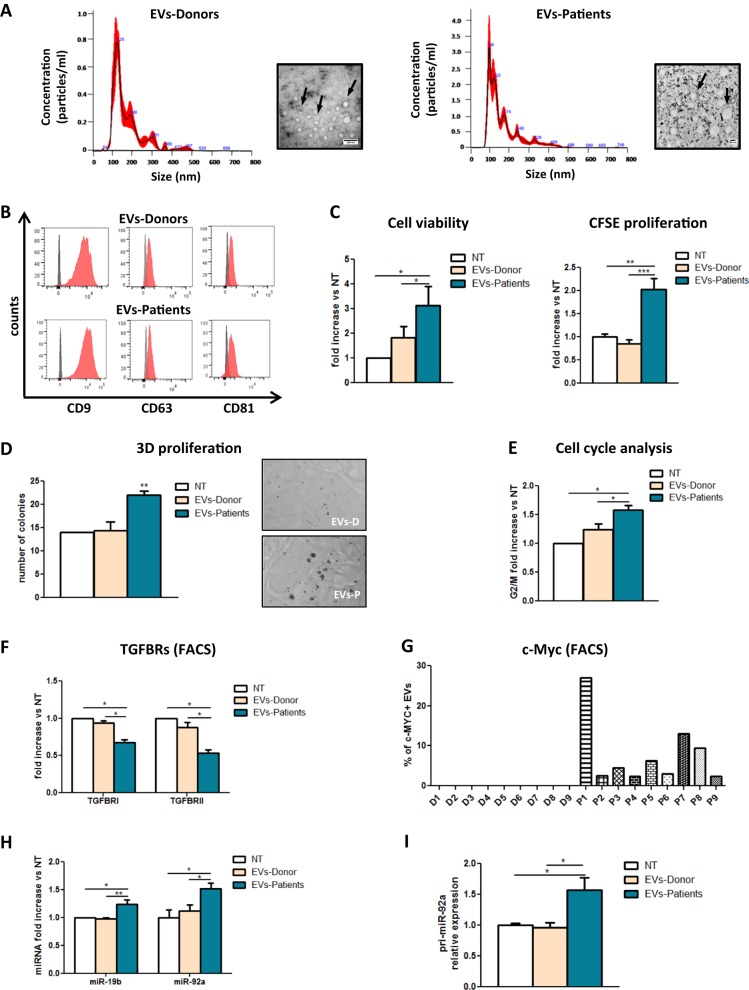
Lung cancer patient EVs increased bronchial epithelial cell proliferation. **a** Concentration and size distribution graphs (NanoSight) and corresponding representative images (TEM) of EVs-Donors (left) and EVs-Patients (right). **b** FACS analysis of the exosomal markers CD9, CD63, CD81 and Alix (representative images, *n* = 3). **c**–**e** Modulation of the HBEC-KRAS^V12high^ cell phenotype after EV treatment. **c** Cell viability (left; *n* = 10) and CFSE proliferation (right; *n* = 10) in cells treated with EVs-Donors and EVs-Patients compared to control-treated cells. **d** Effect of EVs on HBEC-KRAS^V12high^ cell colony formation ability (*n* = 5; Left: quantification of colonies, right: representative images) and on the cell cycle (**e**; n = 6). **f** TGFBRI and TGFBRII protein expression levels in HBEC-KRAS^V12high^ cells treated as previously described (*n* = 8). **g** Graph bars showing the percentages of EVs positive for c-Myc in each sample (EVs derived from 9 donors (D) and 9 patients (P) using FACS). **h**, **i** miR-19b, miR-92a (**h**) and pri-miR-92a (**i**) expression levels in HBEC-KRAS^V12high^ cells after EV treatment compared to no treatment (*n* = 10). Data are expressed as the mean ± SEM. *p < 0.05, **p < 0.01

To verify whether our proposed mechanism reflects in vivo lung cancer patient conditions, we isolated EVs from the plasma of lung cancer patients (EVs-Patients) and heavy smokers (EVs-Donors), and we investigated their functional effects on HBEC-KRAS^V12high^ cells. NanoSight analysis revealed that the EVs-Patients and EVs-Donors had a similar median size (166.9 and 174.7 respectively, Fig. 6a, Supplementary Table 2), with greater size heterogeneity seen in the EVs-Patients (Fig. 6a). Both the EVs-Donors and EVs-Patients were positive for the exosomal markers CD9, CD63, and CD81 (Fig. 6b). The same amount (15 μg) of EVs-Patients and EVs-Donors was added to HBEC-KRAS^V12high^ cells, and we observed an increase in the proliferation rate of the cells treated with the EVs-Patients (2-fold increase, *p* < 0.05) that was not detectable in the cells treated with the EVs-Donors (Fig. 6c, left). CFSE proliferation (Fig. 6c, right, Supplementary Fig. 15), 3D cell culture (Fig. 6d) and cell cycle analyses (Fig. 6e) confirmed these data. Furthermore, we detected downmodulation of both TGFBRI and TGFBRII expression after treatment with EVs-Patients compared to treatment with EVs-Donors (Fig. 6f, Supplementary Figs. 11 and 12). Only the EVs derived from patients contained c-Myc protein, although the percentage varied among individuals (Fig. 6g, Supplementary Fig. 13a). This result suggests that c-Myc is specifically carried by tumoural EVs, while it is absent in EVs derived from heavy-smoker donors. As expected from this result, EVs-Patients but not EVs-Donors induced upmodulation of miR-19b and miR-92a expression in recipient cells (Fig. 6h) as a consequence of the increased transcription of pri-miR-92a (Fig. 6i).

Overall, these data suggest that EVs derived from lung cancer patients can influence the tumour microenvironment and promote epithelial cell proliferation.

## Discussion

It is known that lung cancer patients have an increased risk for a second primary tumour in the lung (0.7–15% of NSCLC patients)^37^ or intrapulmonary metastasis (4% in resected NSCLC)^38^. Our work demonstrated that EVs were able to increase proliferating activity of pre-neoplastic epithelial cells such as HBEC-KRASV12high which could favour tumour growth or modulate lung microenvironment for the establishment of intrapulmonary metastatic niche.

The tumour microenvironment plays a central role in cancer development and progression^39,40^, and in particular, EVs seem to have a crucial role in modulating the tumour microenvironment that directly surrounds the primary tumour or metastatic lesion. EVs affect several key cellular mechanisms, including oncogenic transfer, angiogenesis and pre-metastatic niche formation^41^. In addition, EVs are able to influence multiple aspects of the immune system, such as immunomodulation and cancer immune evasion^42^.

Cancer-derived EVs affect not only cells in the microenvironment but also other tumour or pre-neoplastic cells, resulting in modulation of the tumourigenic potential of the affected cells^43,44^. We demonstrated that EVs derived from two different lung adenocarcinoma cell lines were able to increase the proliferative ability of non-tumourigenic bronchial epithelial cells. Similar to other works in which EVs were shown to modulate the malignant phenotype of epithelial cells^17^, our study showed increases in the proliferation and 3D growth of HBECs after treatment with lung cancer-derived EVs. This pro-tumourigenic modulation was confirmed by the enhancement of the proportion of cells in the G2/M cell cycle phase.

The role of EV-mediated activation of recipient cells through the transfer of molecules such as proteins and miRNAs has also been described by recent studies. In line with the findings of studies that indicated the involvement of miRNAs in malignant cell transformation^45^, our data suggest that the modulation of epithelial cell proliferation is mediated by the upregulation of miR-19b and miR-92a transcription in HBECs. These two miRNAs are members of the miR-17/92 cluster, which is often deregulated in lung cancer and is an important regulator of the cell cycle, proliferation and other cellular processes. Interestingly, cluster members have also been described to cooperate in cell cycle control, particularly in the context of TGFB signalling also in neuroblastoma^29,46^. Moreover, TGFB signalling is deregulated in tumours and influences several processes, such as cell growth, differentiation, apoptosis, motility and immunity^47,48^. We demonstrated that lung cancer EVs increased miR-19b and miR-92a transcription, downmodulating the expression of TGFBRI and TGFBRII in treated HBECs. TGFB receptors are considered tumour suppressor genes in several cancers, and downmodulation of the expression of these receptors promotes carcinogenesis in epithelial tumours^49,50^.

Since we observed an increase in pri-miR-92a mRNA after EV treatment, we hypothesised that there is a transcription factor within EVs responsible for the miR-19b and miR-92a upregulation. The miR-17–92 cluster has been reported to be upregulated by the oncogene c-Myc^36^, which can directly bind to the promoter of the miR-17/92 cluster to initiate transcription. Lobb et al. revealed the presence of this transcription factor in lung cancer exosomes^51^, suggesting the potential for c-Myc to be transferred from exosomes to HBECs to increase cell proliferation. Moreover, Sato et al.^28^ found that the overexpression of c-Myc greatly enhanced malignancy in HBEC-sh-p53 + KRAS^V12^ cells and induced EMT and in vivo tumour growth. Our data demonstrate how EVs secreted by tumour cells can modify the epithelial tumour microenvironment through the transfer of molecules to modulate the malignant phenotype of normal epithelial cells, with particular associations with proliferation and TGFB signalling regulation.

We clearly demonstrated that the pro-tumourigenic effects of EVs isolated from lung cancer cell lines were also observed using lung cancer patient-derived EVs, mimicking what could happen in the lung cancer microenvironment in patients.

Interestingly, for the first time, we detected the presence of c-Myc inside EVs isolated from lung cancer cells and EVs isolated from the plasma of lung cancer patients. We showed that these EVs have the ability to induce premalignant changes in recipient HBEC-KRAS^V12high^ cells, suggesting a potential EV-mediated pro-tumourigenic mechanism that could occur in lung cancer patients.

However, whether the effect of EVs on lung epithelial cells mediated through miRNA modulation is a process limited to lung cancer or is a broader mechanism has yet to be unveiled. Our results set the basis for further analysis of the role of EVs in the plasma as biomarkers or as therapeutic tools in lung cancer.

## Materials and methods

### Cell lines and treatments

The human lung adenocarcinoma cell line A549 was purchased from the American Type Culture Collection (ATCC; LGC Standards), whereas the LT73 cell line was derived in our laboratory from a primary lung tumour in a 68-year-old male. Both cell lines were cultured in RPMI 1640 medium (Gibco, Thermo Fisher Scientific, Waltham, MA, USA) supplemented with 10% foetal bovine serum (FBS; EuroClone, Italy) and 1% penicillin-streptomycin (Sigma-Aldrich, Saint Louis, MO, USA).

Immortalised bronchial epithelial cells with different genetic alterations (HBEC-KRAS^V12high^ cells: hTERT + Cdk4 + sh-p53 + KRAS^V12high^) were provided by Prof. J. D. Minna^28^ at the University of Texas Southwestern Medical Center. HBECs were maintained in K-SFM medium (Thermo Fisher Scientific) supplemented with 5 ng/mL human recombinant EGF and 50 μg/mL bovine pituitary extract (Thermo Fisher Scientific). For EV isolation, complete medium was replaced with serum-free medium 48 h prior to isolation. Cell lines were cultured in a humidified incubator containing 5% CO_2_ at 37 °C. All cell lines were authenticated by DNA short tandem repeat (STR) profiling and confirmed to be mycoplasma negative.

In total, 5 × 10^4^ HBEC-KRAS^V12high^ cells were seeded in a 6-well plate and treated with tumour-derived EVs (15 μg/well) or transfected with miR-19b-3p (miR-19b) or miR-92a-3p (miR-92a) mimics or a negative control (scr) (5 nM, Thermo Fisher Scientific) using Lipofectamine 2000 (Thermo Fisher Scientific) following the manufacturer’s protocols. Transfection of the appropriate LNA inhibitor (50 nM; Exiqon, Vedbæk, Denmark) was performed 24 h before EV or EV-depleted CM treatment (indicated as LNA EVs and LNA CM-EVs depleted, respectively), as described above. A human MYC plasmid construct was purchased from OriGene (SC112715, pCMV6-XL5-MYC; OriGene Technologies, Rockville, MD, USA) and transiently transfected into HBEC-KRAS^V12high^ cells following the company’s protocol. Cells were collected at 24 or 72 h after treatment.

### Clinical specimens

Plasma samples were collected from high-risk heavy-smoker volunteers aged 50 to 75 years old, including current or former smokers with a minimum pack/year index of 30 enroled in a low-dose computed tomography (LDCT) screening trial performed at our institution (BioMild Trial)^52^, and from lung cancer patients in the Istituto Nazionale Tumori (INT)-Thoracic Unit (Supplementary Table 1). Specimen collection was approved by the Internal Review and the Ethics Boards of the INT of Milan. All patients provided informed consent.

### EV isolation

EVs from CM and plasma were purified by differential centrifugation processes, as shown in Supplementary Fig. 16. For CM-derived EV isolation, 1 × 10^6^ cells/ml were cultured in 175-cm^2^ flasks for 48 h with serum-free medium. CM was collected and centrifuged at 300 x g for 10 min and then at 3200 × *g* for 25 min to remove residual cells and debris. To exclude large vesicles, the supernatant was filtered through 0.22-μm filters (Millipore, Burlington, MA, USA) and then ultracentrifuged at 120,000 × *g* for 90 min at 4 °C using a TLA-100.3 fixed-angle rotor in a TL-100 ultracentrifuge (Beckman Coulter, Brea, CA, USA). The resulting supernatant was collected and stored at −80 °C as CM-EV depleted while the EV-enriched pellet was washed in phosphate-buffered saline (PBS; Thermo Fisher Scientific) at the same ultracentrifuge speed for 60 min at 4 °C. Then the pellet was resuspended in PBS or directly lysed in RIPA buffer (Sigma-Aldrich) with protease and phosphatase inhibitors and stored at −80 °C. The protein content of the purified EVs was determined by the Bradford assay. Regarding plasma-derived EVs, plasma was separated from whole blood as described in Fortunato et al.^53^. EV isolation was performed by ultracentrifugation starting with 1 ml of stored plasma, as described above and shown in Supplementary Fig. 16.

The EV concentration and size distribution were determined by using a NanoSight NS300 instrument (Malvern Panalytical). Five 30-s videos were recorded for each sample with a camera level set at 15/16 and a detection threshold set between 2 and 7. The videos were subsequently analysed with NTA 3.2 software to calculate the size and concentration of the particles. Auto settings were used for the analysis.

### TEM

EV morphology was measured using a Zeiss LIBRA 200FE transmission electron microscope with an in-column second-generation omega filter. Samples were prepared as follows: a suspension drop (7 μl) was placed on a TEM copper grid covered with a carbon/formvard film. After blotting, a negative staining procedure was performed using UranyLess (EMS-Electron Microscopy Science), a contrast agent^54^. The estimation of EV size was performed by measuring a hundred EVs using the iTEM-TEM Imaging platform (Olympus).

### Western blotting

Cells and EV pellets were lysed in RIPA buffer. Then, 40 µg of protein lysate was loaded on a Bolt 4–12% Bis-Tris gel (Thermo Fisher Scientific). Western blot analyses were performed using the following antibodies: anti-CD9 (Cell Signaling; 1:1000), anti-CD81 (Thermo Fisher Scientific; 1:100) and anti-Alix (BioLegend; 1:1000), c-Myc (Cell Signaling, 1:1000) primary antibodies and the corresponding anti-mouse and anti-rabbit peroxidase-linked secondary antibodies (GE Healthcare Life Sciences, 1:2000). Signal detection was performed via chemiluminescence reaction (ECL, GE Healthcare) using the MINI HD9 Western Blot Imaging System (Cleaver Scientific Ltd., United Kingdom). Western blot quantification was performed using ImageJ software analysis.

### Flow cytometry analysis

Flow cytometry analysis of EVs was performed as previously described^55^, starting with 30 μg of EVs. Briefly, we used 1 µg each of primary anti-CD9, anti-CD81, anti-CD63 (Abcam, Cambridge, UK), and anti-c-Myc (Cell Signaling, Danvers, Massachusetts, USA) antibodies and the corresponding fluorescent secondary antibodies (Alexa Fluor 488-conjugated goat anti-rabbit IgG, Thermo Fisher Scientific; Dylight 488-conjugated goat anti-mouse IgG, Bethyl), both incubated for 30 min at 4 °C. For c-Myc analysis, EVs were permeabilized with a 0.1% Triton solution (15 min, room temperature (RT)) prior to incubation with a primary Ab.

TGFBRI analysis was performed with a primary anti-hTGFBRI antibody (Abcam; 1:100) and secondary Alexa Fluor 488-conjugated goat anti-rabbit antibody (Thermo Fisher Scientific; 1:2000) incubated for 1 h at 4 °C. For TGFBRII evaluation, we used a FITC-conjugated anti-hTGFBRII antibody (R&D Systems, Minneapolis, MN, USA; 1:20) incubated for 15 min at RT.

All analyses were performed by flow cytometry (FACSCalibur, BD Biosciences, San Jose, CA, USA) and used FlowJo software (TreeStar, Ashland, OR, USA). For c-Myc analysis, we fixed the threshold of positivity at 1%: samples with the percentage of c-MYC^+^-exosomes below 1% were classified as negative.

### Enzyme-linked immunosorbent assay

c‐Myc (total) human ELISA kit (ThermoFisher Scientific) was used to measure the level of c‐Myc in cellular and EVs-derived lysates, whereas RayBio Human c-Myc (MYC) ELISA Kit for cell culture supernatant samples. All tests were performed in triplicate. Absorbance at 450 nm was measured using a microplate reader (Infinite M1000, Tecan).

### miRNA and mRNA expression analyses

MiRNA levels were determined using chip-based digital PCR (dPCR) (Thermo Fisher Scientific) as previously described^56^. Briefly, total RNA was extracted using the Maxwell RSC Instrument (Promega, Madison, WI, USA) and Maxwell RSC miRNA Tissue Kit (Promega) following the manufacturer’s instructions. Synthetic *Caenorhabditis elegans* miRNA-39 (cel-miR-39) was used as a spiked-in control and was added to each sample at a concentration of 25 fmol from the stock tube (Qiagen, Hilden, Germany)^57^. Total RNA (20 ng) was reverse transcribed into cDNA using an RT primer pool specific for the miRNAs of interest. A pre-amplification step was performed using 2.5 µl of RT product and a Custom TaqMan PreAmp primer pool (Thermo Fisher Scientific). MiRNA expression was evaluated in 2.5 µl of PreAmp product using dPCR.

For analysis of EV miRNA content, 15 μg of EVs were treated with RNAse A (10 µg/ml; Roche, Basel, Switzerland) for 30 min to eliminate miRNAs present outside the exosomes. Then, total RNA was extracted and analysed as described above.

Gene expression analysis was assessed by reverse transcription starting with 250 ng of total RNA. TaqMan Gene Expression Assays (Thermo Fisher Scientific) and the Applied Biosystems 7900 System (Thermo Fisher Scientific) were utilised to quantify and analyse the levels of selected genes. The B2M gene was used as a reference for sample normalisation.

### EV labelling and internalisation

EVs were labelled with PKH67 Fluorescent Cell Linker kits (Sigma-Aldrich) as follows: EV pellets were incubated with 50 μl of PKH67 dye diluted in Diluent C (Sigma-Aldrich; 1:1000) for 5 min at RT. To eliminate excess dye, the pellet was washed with 1 ml of PBS, ultracentrifuged at 120,000 × *g* for 60 min at 4 °C and then resuspended in PBS.

For confocal microscopy analysis, PKH67-labelled EVs were added to 5 × 10^4^ HBEC-KRAS^V12high^ cells grown on a coverslip. After 24 h, the cells were fixed for 15 min in a flow cytometry fixation buffer (1X; R&D Systems) and washed with PBS. Then, the cell membranes were stained with Alexa Fluor 633-conjugated wheat germ agglutinin (WGA) (1:2000, 1 mg/ml stock solution; Thermo Fisher Scientific) for 10 min at RT. The nuclei were stained with DAPI (1:1,500, 5 mg/ml stock solution; Life Technologies) for 10 min at RT, and the samples were mounted with ProLong Gold Antifade reagent (Thermo Fisher Scientific). A confocal laser scanning microscope (Leica TCS SP8 X) was used to visualise the samples, and Leica LAS X rel. 2.0.1 software (Leica Microsystems, Mannheim, Germany) was used to acquire images. Each fluorophore was excited independently, and sequential detection was performed. Each image consisted of a z-series of images (step size, 0.5 µm) with 512 × 512 pixel resolution and was reported as extended depth of field using an HC PL APO 63X/1.40 CS2 oil immersion objective and a pinhole set to 1 Airy unit. The obtained data were analysed using ImageJ software.

For FACS analysis, HBEC-KRAS^V12high^ cells were labelled with PKH26 dye (Sigma-Aldrich) according to the manufacturer’s instructions and cultured with PKH67-labelled EVs. In brief, the HBEC-KRAS^V12high^ cells were resuspended in 1 ml of Diluent C, and 4 μl of PKH26 dye was diluted in 1 ml of Diluent C. The solutions were mixed and incubated for 5 min, with the subsequent addition of 2 ml of FBS to bind any excess PKH26 dye. The labelled HBEC-KRAS^V12high^ cells were centrifuged and then seeded in a 6-well plate at 5 × 10^4^ cells/well. The following day, the cells were incubated with the PKH67-labelled EVs. All the analyses were performed using a FACSCalibur and FlowJo software.

### Functional assays

Viable cells were counted after 72 h of treatment using a Trypan blue solution (Sigma-Aldrich). Proliferation was evaluated with a CFSE assay (Thermo Fisher Scientific) following the manufacturer’s protocol.

For cell cycle analysis, HBEC-KRAS^V12high^ cells were harvested, washed with ice-cold PBS and fixed overnight with 70% ethanol at 4 °C. After being washed twice with ice-cold PBS, the cells were incubated with a propidium iodide (PI) solution (50 μg/ml, 0.1% Triton X100 and 0.1% sodium citrate) containing RNAse A (0.5 mg/ml) (Roche) at 37 °C for 40 min in the dark. The stained cells were analysed by a FACSCalibur and FlowJo software.

A 3D proliferation assay was performed using VitroGel 3D (The well Bioscience, USA) following the manufacturer’s instructions. Briefly, a 1:1 VitroGel mix was added into 24-well plates. In total, 5 × 10^4^ HBEC-KRAS^V12high^ cells were plated on top of the VitroGel, and colonies were counted after 72 h of treatment. Five random fields were counted for each condition.

Migration was evaluated by a wound healing assay using the JuLI Stage Instrument (NanoEnTek Inc.) and a JuLI Stage Real-Time Cell History Recorder tool.

To confirm the specific role of EVs in functional studies and exclude the potential involvement of other factors released by cells into CM, we used treatment with EV-depleted CM as a negative control. Among the tested dilutions, we observed that the 1:4 dilution in K-SFM medium was useful for meeting the mentioned criteria (Supplementary Fig. 17). Untreated cells were used as an additional control.

### miRNA target prediction

Computational miRNA target prediction analysis was performed using the DIANA tool and starBase v3.0 platform. In detail, DIANA-mirPath v3 and miRTarPathway-starBase were used to identify molecular pathways potentially altered by miRNAs, while DIANA-TarBase v8 and the PITA programme were used for gene enrichment analysis of experimentally validated miRNA target genes.

The sequences of miR-19b-3p and miR-92a-3p were extracted from the miRBase database (miR-19b-3p miRBase ID: MIMAT0000074; miR-92a-3p miRBase ID: MIMAT0000092).

### Luciferase reporter assays

The pLightSwitch™ 3′UTR Reporter vector containing the 3′UTR of TGFBRII was purchased from Active Motif (ID S811544; Active Motif, Carlsbad, CA USA). A 3′UTR fragment (468 bp) from TGBRI containing the predicted target site for miR-92a was PCR amplified from cDNA produced from HBEC-KRAS^V12high^ cells using the following primers:

*Fw 5*′*-GGGGCTAGCAACTCTGCTGTGCTGGAGATC-3*′

*Rev 5*′*-GGGCTCGAGCAAAACAGAAAAAGTTTGGGTTACCC-3*′

and cloned into the pLightSwitchTM vector (ID S890005, Active Motif) between the NheI and XhoI restriction sites. Correct insertion of the TGFBRI 3′UTR was confirmed by sequencing analysis (Eurofins Genomics, Ebersberg, Germany). Predicted target sites for miR-19b and miR-92a were mutated by direct mutagenesis of the pLightSwitch 3′UTR vectors, using the PCR-based QuikChange II site-directed mutagenesis kit (Agilent Technologies, CA, USA) according to the manufacturer’s instructions and the following primers:

*TGFBRI-3*′*UTR-Fw 5*′*-GGAACATAATTCATAAGGCTGTATTTTGTATAC-3*′

*TGFBRI-3*′*UTR-Rev 5*′*-GTATACAAAATACAGCCTTATGAATTATGTTCC-3*′

*TGFBRII-3*′*UTR-Fw 5*′*-CAATAGCCAATAACAGGGTTACTTTATTAATGCC-3*′

*TGFBRII-3*′*UTR-Rev5*′*-GGCATTAATAAAGTAACCCTGTTATTGGCTATTG-3*′

The presence of the mutations was confirmed by sequencing (Eurofins Genomics). The luciferase constructs were transfected into HEK293 cells together with miR-19b, miR-92a or a scrambled oligonucleotide sequence. The cells were cultured for 48 h and assayed with the Luciferase Reporter Assay System (Active Motif, Carlsbad, CA USA) according to the manufacturer’s instructions.

### Statistical analyses

All experiments were performed at least in triplicate, and all values are reported as the mean ± SEM. Analyses were performed using GraphPad Prism (GraphPad Software, La Jolla, California USA). Intergroup comparisons were assessed by a two-tailed Student’s *t*-test, and a *p*-value <0.05 was considered statistically significant. Statistical significance has been indicated as follows: *p < 0.05, **p < 0.01, and ***p < 0.001.

## Supplementary information

Supplementary material

Supplementary Figure
