# Supplementary material for: c-Myc shuttled by tumour-derived extracellular vesicles promotes lung bronchial cell proliferation through miR-19b and miR-92a
Source: Cell Death Dis. 2019 Oct 7;10(10):759. doi: 10.1038/s41419-019-2003-5 (PMC6779734; doi:10.1038/s41419-019-2003-5)
Supplement: Supplementary file 1 — Supplementary material [file 41419_2019_2003_MOESM1_ESM.docx]

**SUPPLEMENTARY FIGURE LEGENDS**

**Suppl. Figure 1. EVs characterization**. (**A**) Size distribution of HBEC-KRAS^V12high^-derived EVs, obtained by NanoSight. (**B**) Total protein content of EVs isolated from different lung cell lines (HBECs, A549, LT73), starting from the same amount of cells (n = 3) (**C**) Frequencies of mean diameter (d_m_; nm) of EVs isolated from A549 and LT73. (**D**) Representative images (*left*) and frequencies of mean diameter (d_m_; nm) (*right*) of HBEC-KRAS^V12high^ derived EVs, obtained by TEM. Yellow arrows indicate EVs, red arrows indicate artefacts. (**E**) FACS analysis of exosomes markers CD9, CD63 and CD81 in HBEC-KRAS^V12high^ (representative images, n = 3).

**Suppl. Figure 2.** **EV uptake by recipient cells.** FACS analysis of PKH26-labeled HBEC-KRAS^V12high^ cultured with PKH67-labeled EVs for 24, 48 and 72 h. *Left*: graphs show fluorescence of EVs at the indicated time points; *right*: representative images of HBEC-KRAS^V12high^ labelled signal, detected at 72 h.

**Suppl. Figure 3.** **CFSE proliferation analysis in EVs-treated cells.** Examples of CFSE proliferation analysis using FACS. Cells were treated as indicated in figure.

**Suppl. Figure 4**. **EVs did not affect migration and epithelial-to-mesenchymal transition** (**A**) HBEC-KRAS^V12high^ migration ability after the indicated treatments (wound healing assay, n = 3). *Left*: relative wound closure time compared to untreated cells; right: time course of wound closure (%) in the different conditions. (**B**) Expression level of genes involved in EMT process (qPCR, n = 3). B2m was used as housekeeping control. In all experiments, untreated cells were used as control. Data are expressed as mean ± SEM.

**Suppl. Figure 5. miRNAs modulation in EVs treated HBEC-KRAS^V12high^.** miRNAs expression levels (copies/μl) in HBEC-KRAS^V12high^ after 24 h of treatment with EVs derived from A549 (*red bars*) or LT73 (*green bars*) (n = 2). Data are expressed as mean ± SEM.

**Suppl. Figure 6. EVs-driven phenotypic modulation of HBEC-KRAS^V12high^ was not affected by miR-19b and miR-92a EVs depletion.** (**A**) miR-19b and miR-92a expression level in EVs-derived from A549 treated with miR-19b and miR-92a inhibitors (LNA-19b and LNA-92a). (**B-D**) Cell, viability, proliferation and cell cycle (**B**), miR-19b and miR-92a expression level (**C**), and TGFBRs fold increase (**D**) in HBEC-KRAS^V12high^ upon exposure to EVs derived from A549(LNA). (**E**) Percentage of c-Myc+ EVs in A549 WT- and A549(LNA)-derived EVs. Data are expressed as mean ± SEM. **p < 0.05, **p < 0.01, ***p<0.001, ****p < 0.0001.*

**Suppl. Figure 7. *In vitro* miR-19b and miR-92a overexpression in HBEC-KRAS^V12high^ cells reproduced miRNAs increase induced by EVs exposure.** (**A**) miR-19b and miR-92a relative expression level in HBEC-KRAS^V12high^ upon treatment with the corresponding miRNAs-mimics (qPCR, n = 3). (**B**) Comparison of miR-19b and miR-92a expression level (copies/µl) between HBECs treated with miRNAs mimic (*white bar*) or EVs (*green bars*).

**Suppl. Figure 8. miR-19b and miR-92a inhibition in recipient cells reverted miRNAs modulation after EVs treatment.** miR-19b (l*eft*) and miR-92a (*right*) relative expression level in HBEC-KRAS^V12high^ 72 h after treatment with CM-EVs depleted or EVs and corresponding miRNA inhibitor (LNA-19b and LNA-92a, respectively,) compared to LNA NT ( n = 3). Data are expressed as mean ± SEM. **p < 0.05, **p < 0.0,* **********p < 0.001*

**Suppl. Figure 9. HBEC-KRAS^V12high^ derived EVs did not reproduce the phenotypic modulation induced by tumour cells-derived EVs in the recipient cells.** (**A**) Cell viability, (**B**) CFSE and (**C**) cell cycle analysis of HBEC-KRAS^V12high^ treated with 15 µg of EVs derived from HBEC-KRAS^V12high^ (n = 3). (**D**) Pri-miR-92a (qPCR, n = 3) and (**E**) TGFBRs expression levels (FACS, *left*, and qPCR, *right*, n = 3) after the indicated treatments. B2m was used as housekeeping control. Data are expressed as mean ± SEM.

**Suppl. Figure 10. Transcriptional regulation of TGFBR in HBEC-KRAS^V12high^ treated cells.** (**A**) TGFBRI (*left*) and TGFBRII (*right*) mRNA expression level in EVs treated cells (n = 3). (**B**) Modulation of TGFBRI and TGFBRII transcripts following miR-19b and miR-92a over-expression in HBEC-KRAS^V12high^ (n = 3). (**C**)Expression level of gene downstream of TGFBRs activation (qPCR, n = 4) in HBEC-KRAS^V12high^ exposed to tumour-derived EVs. B2m was used as housekeeping control. Analysis was performed at 72 h of treatment. Data are expressed as mean ± SEM. **p < 0.05, **p < 0.01, ***p < 0.001.*

**Suppl. Figure 11. TGFBRI protein expression on HBEC-KRAS^V12high^cells.** Representative images of TGFBRI FACS analysis of HBEC-KRAS^V12high^ treated as indicated.

**Suppl. Figure 12. TGFBRII protein expression on HBEC-KRAS^V12high^cells.** Representative images of TGFBRII FACS analysis of HBEC-KRAS^V12high^ treated as indicated.

**Suppl. Figure 13. EVs-mediated c-Myc transfer to recipient cells.** (**A**) Representative images of c-Myc FACS analysis of the indicated EVs (EVs derived from HBEC-KRAS^V12high^, LT73, A549, A549(LNA) cells and from plasma of donor and lung cancer patients). (**B**) Western Blot of c-Myc protein level in A549 and LT73 (*left*) and HBEC-KRAS^V12high^ (*right*) cellular lysate and in the corresponding EVs. Actin was used as control**.** (**C**) Intracellular c-Myc expression level (pg/µl) in A549 (*green bar*) and LT73 (*red bar*), quantified by ELISA (n=2). (**D**) Expression level of c-Myc regulated miRNAs (dPCR, n = 3) in HBEC-KRAS^V12high^ exposed to tumour-derived EVs. Data are expressed as mean ± SEM. **p < 0.05, **p < 0.01, ***p < 0.001.*

**Suppl. Figure 14.** **CFSE proliferation analysis in c-MYC over-expressing HBEC-KRAS^V12high^.** Graphs show FACS analysis of the CFSE-proliferation assay performed on HBEC-KRAS^V12high^ transfected with c-Myc plasmid.

**Suppl. Figure 15.** **CFSE proliferation analysis of epithelial cells treated with EVs-Donor and EVs-Patient.** Representative images of FACS analysis relating to CFSE proliferation assay of HBEC-KRAS^V12high^ treated with EVs-Donor or EVs-Patient.

**Suppl. Figure 16. Scheme of EVs isolation from conditioned medium and plasma using differential centrifugation method.**

**Suppl. Figure 17**. **CM-EVs depleted as negative control.** Proliferation of HBEC-KRAS^V12high^ cultured for 72 h with conditioned medium-EVs depleted (CM-EV depleted) diluted in K-SFM medium as indicated (n = 3). Data are expressed as mean ± SEM.

**Suppl. Table 1. Characteristics of enrolled subjects.**

|  | **Heavy-smokers (n = 10)** | **Lung cancer patients**  **(n = 10)** |
| --- | --- | --- |
| **Gender** |  |  |
| Male | 8 (80%) | 6 (60.0%) |
| Female | 2 (20%) | 4 (40%) |
| **Age** (years) | 62.8 ± 6.7 | 66.4 ± 7.6 |
| **Smoking habit** (Pack-Year index) | 38.9 ± 7.6 | 40.5 ± 7.4 |
| **Histotype** |  |  |
| ADC | / | 8 (80.0%) |
| SCC | / | 0 (0%) |
| other | / | 2 (20%) |
| **Stage** | / |  |
| Ia-Ib | / | 4 (40.0%) |
| II-III-IV | / | 4(40.0%) |

**Suppl. Table 2.** **Size and concentration of EVs isolated from plasma of a heavy-smoker volunteer (EVs-Donor) and a lung cancer patient (EVs-Patient)**.

Nanosight analysis was performed using 5 µg of EVs from each sample

|  | **EVs-Donor** | **EVs-Patient** |
| --- | --- | --- |
| **Mean size (nm)** | 174.7 ± 8.3 | 166.9 ± 2.8 |
| **Mode size (nm)** | 122.7 ± 5.7 | 104.8 ± 5.8 |
| **Particles/ml** | 6.1x10^9^ ± 4.4x10^8^ | 2.3x10^10^ ± 1.1x10^9^ |

**AUTHOR CONTRIBUTIONS**

C.B., Li.C., O.F. designed the study; acquired, analysed and interpreted the data; and drafted the manuscript. A.M. F. and La.C. acquired and analysed the data. G.S., U.P. designed the study, interpreted the data, and critically revised the manuscript. All authors approved the final version of the manuscript.

C.B., Li.C. and O.F. contributed to the realisation of all the figures. A.M.F. took and analysed TEM pictures and prepared Figures 1, 6 and Suppl. Figure 1. La.C. designed Figures 4.
