# Supplementary material for: c-Myc shuttled by tumour-derived extracellular vesicles promotes lung bronchial cell proliferation through miR-19b and miR-92a
Source: Cell Death Dis. 2019 Oct 7;10(10):759. doi: 10.1038/s41419-019-2003-5 (PMC6779734; doi:10.1038/s41419-019-2003-5)
Supplement: Supplementary file 2 — Supplementary Figure [file 41419_2019_2003_MOESM2_ESM.pptx]

## Slide 1
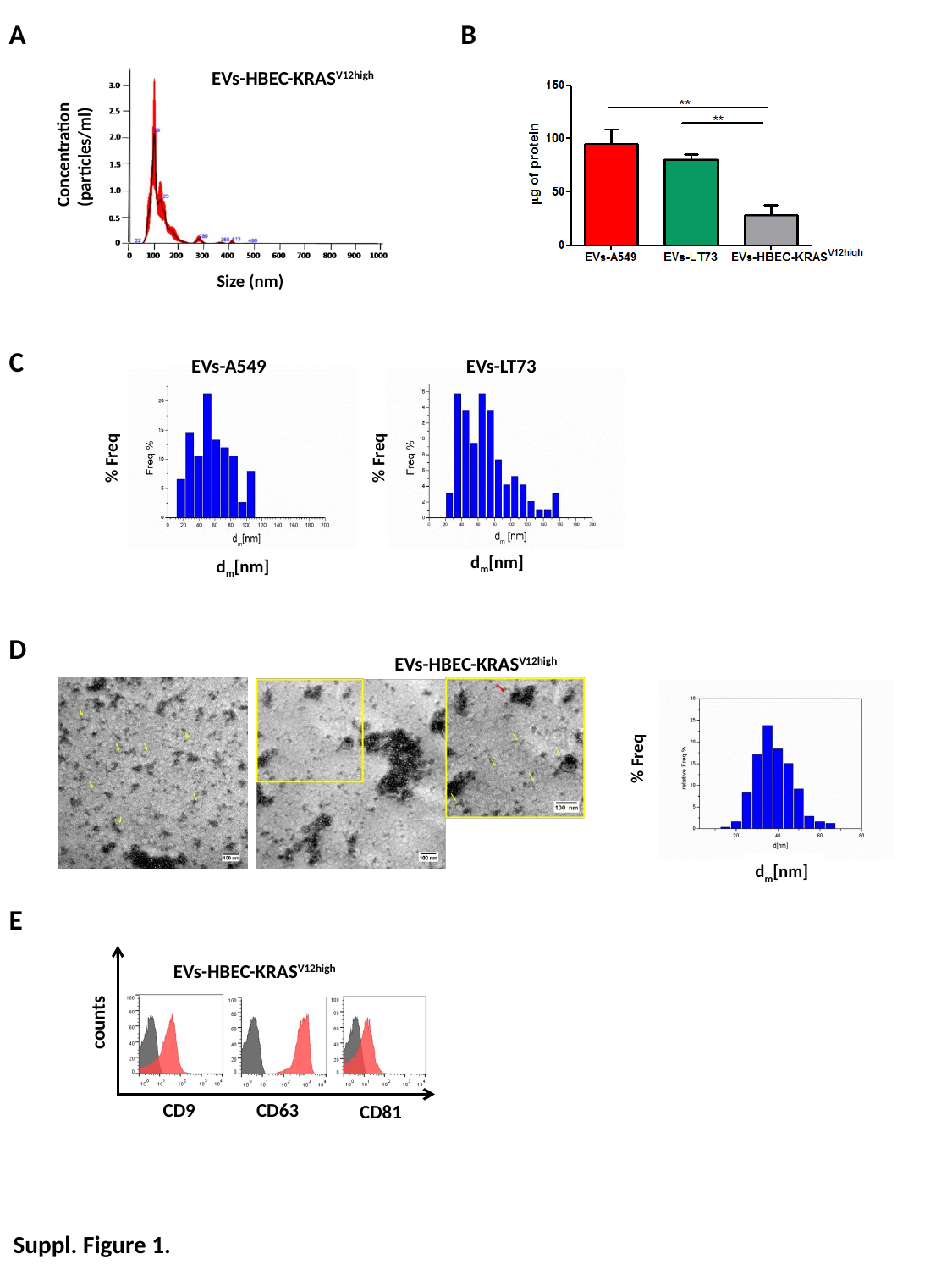

A
B
EVs-HBEC-KRASV12high
Concentration
(particles/ml)
Size (nm)
C
EVs-A549
% Freq
dm[nm]
EVs-LT73
% Freq
dm[nm]
D
EVs-HBEC-KRASV12high
% Freq
dm[nm]
E
EVs-HBEC-KRASV12high
counts
CD63
CD9
CD81
Suppl. Figure 1.

## Slide 2
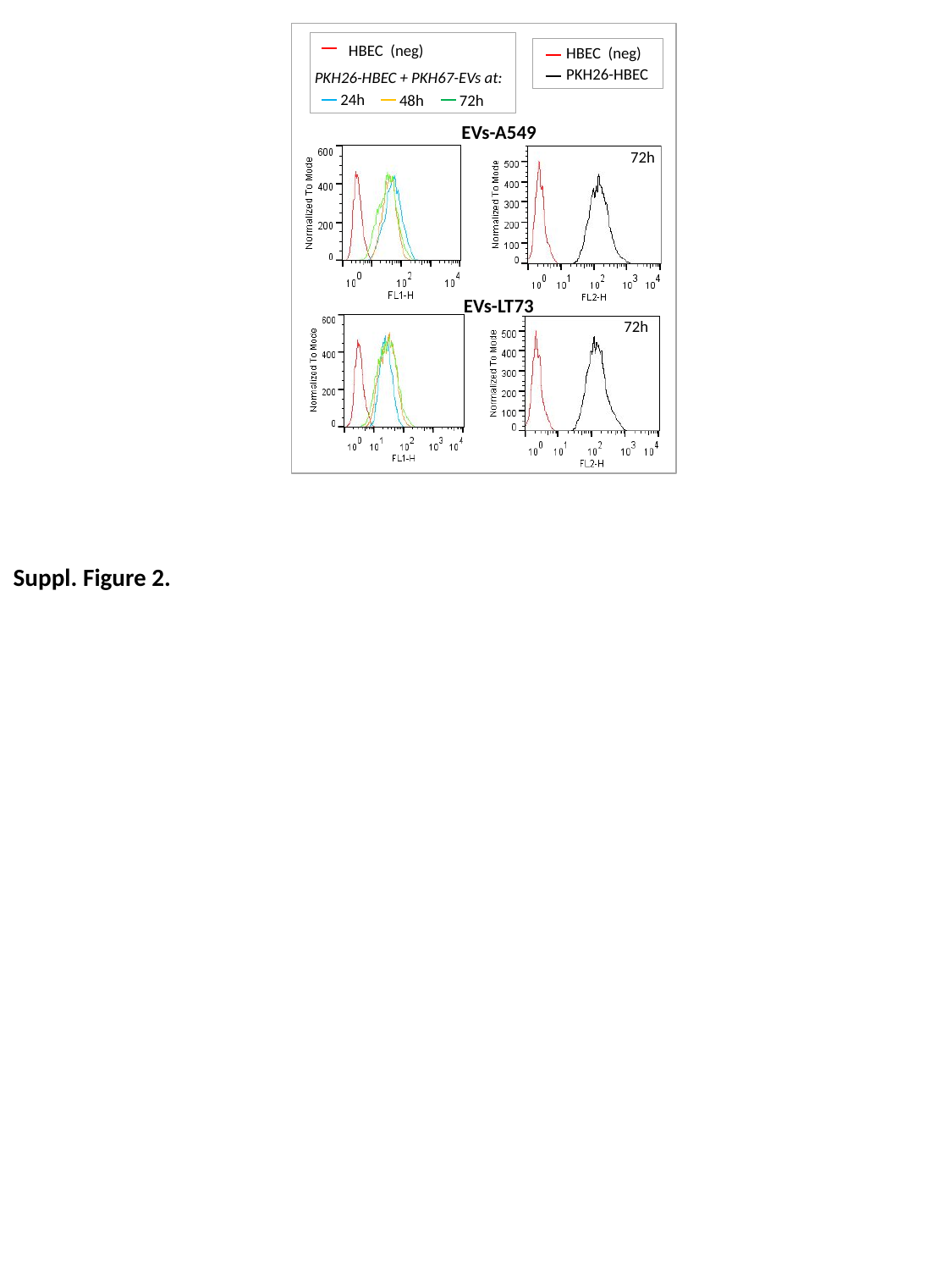

HBEC (neg)
PKH26-HBEC + PKH67-EVs at:
24h
48h
72h
HBEC (neg)
PKH26-HBEC
EVs-A549
72h
EVs-LT73
72h
Suppl. Figure 2.

## Slide 3
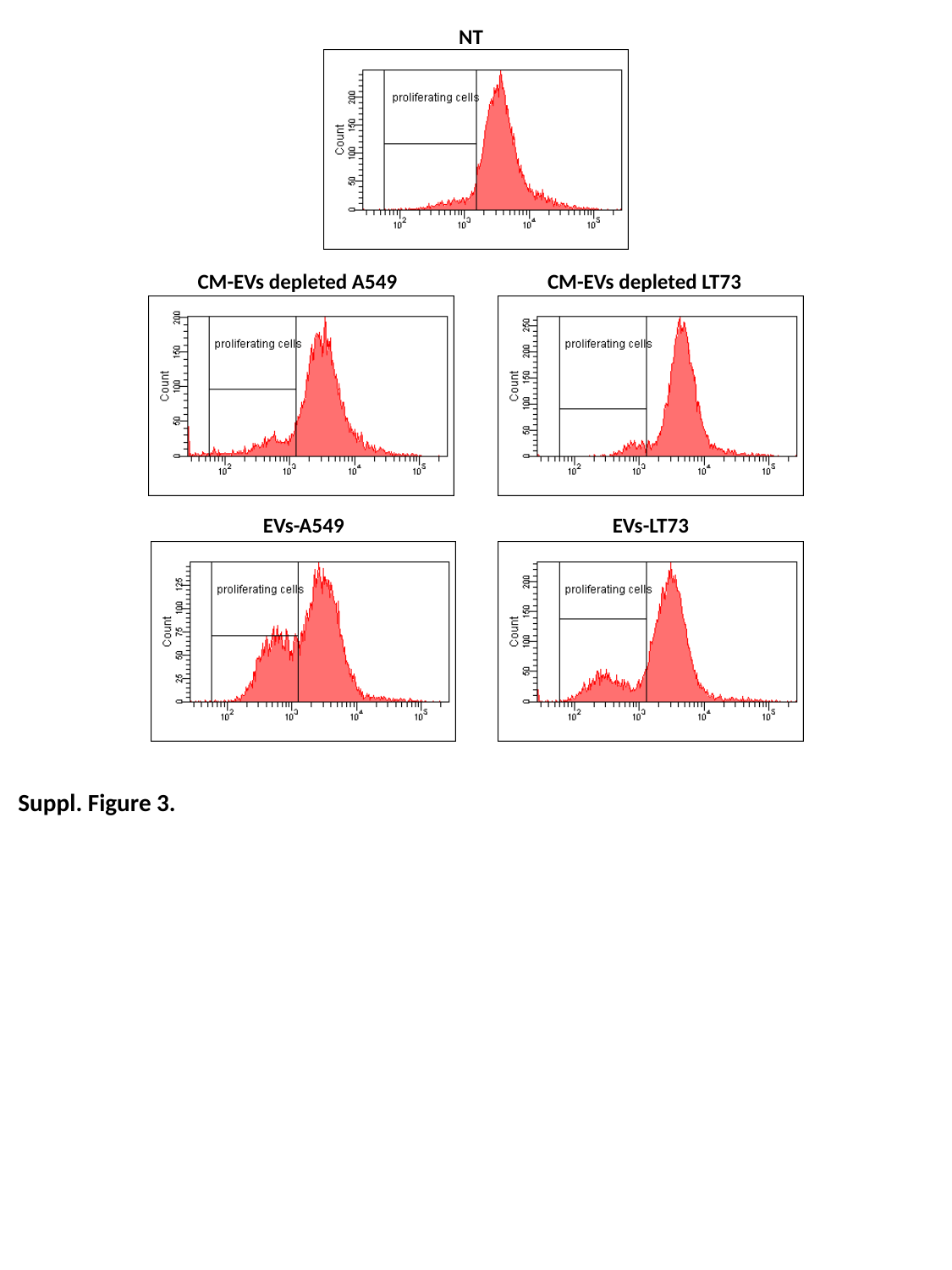

NT
CM-EVs depleted A549
CM-EVs depleted LT73
EVs-A549
EVs-LT73
Suppl. Figure 3.

## Slide 4
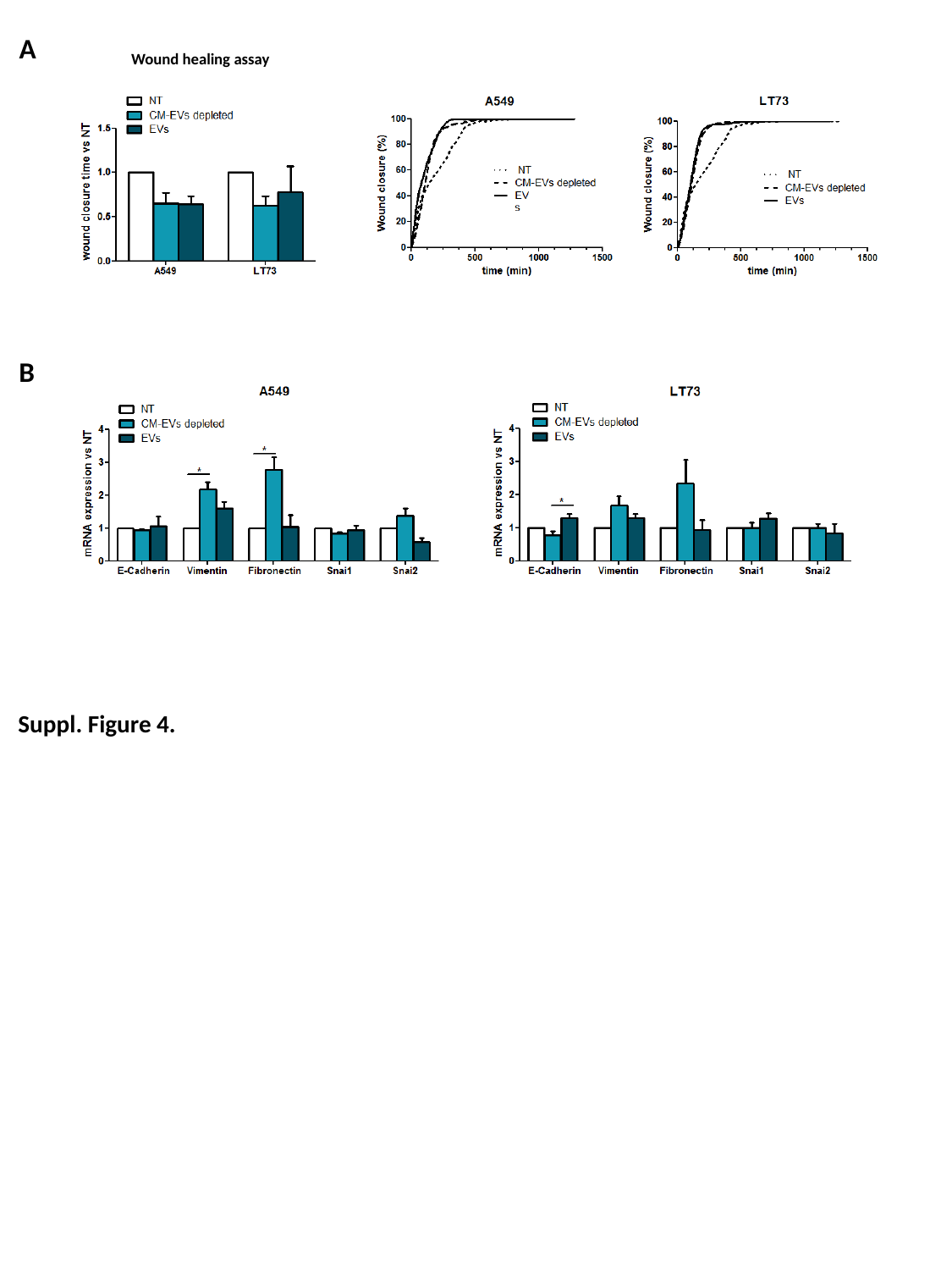

A
Wound healing assay
B
Suppl. Figure 4.

## Slide 5
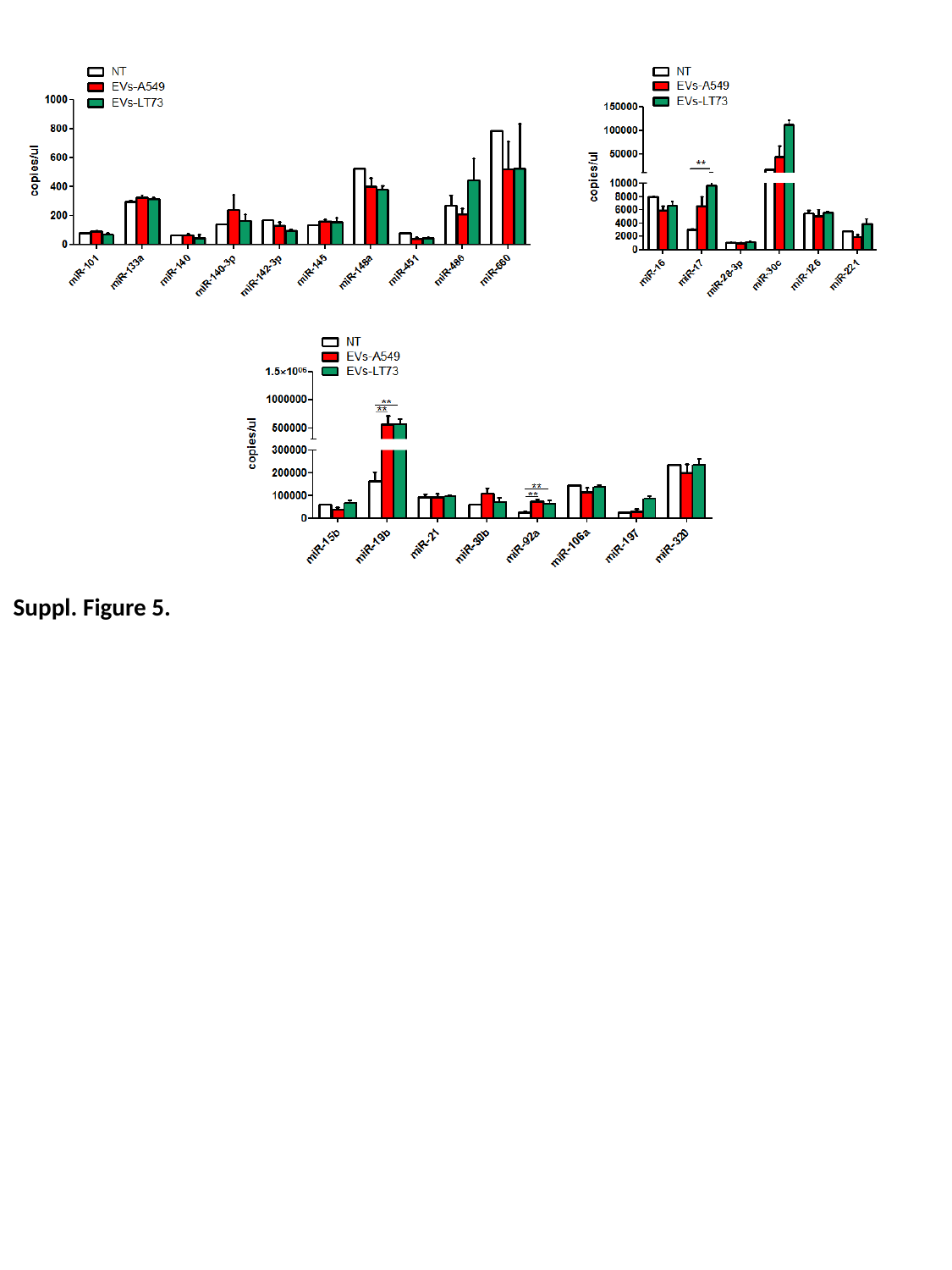

Suppl. Figure 5.

## Slide 6
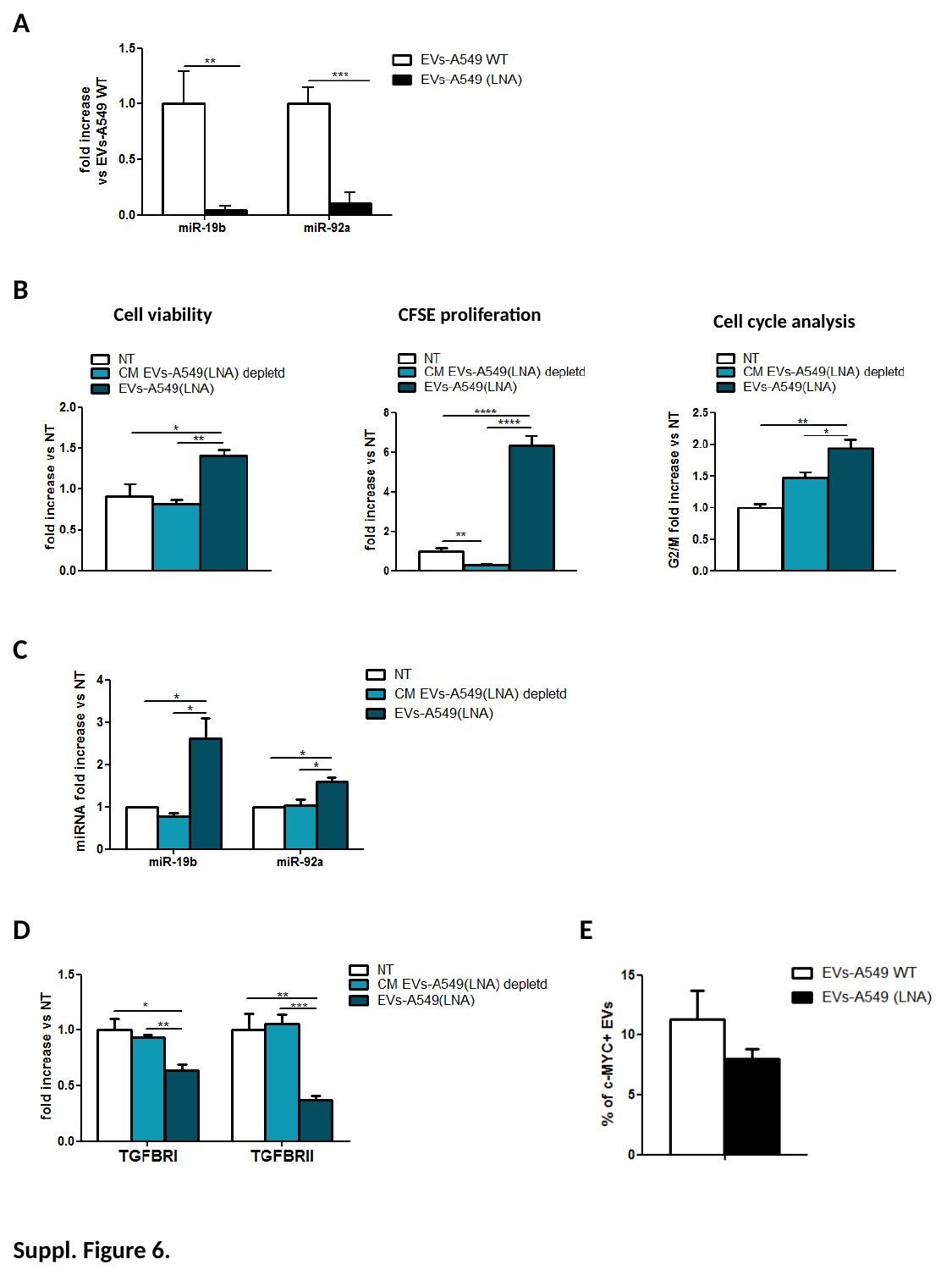

A
B
Cell viability
CFSE proliferation
Cell cycle analysis
C
D
E
Suppl. Figure 6.

## Slide 7
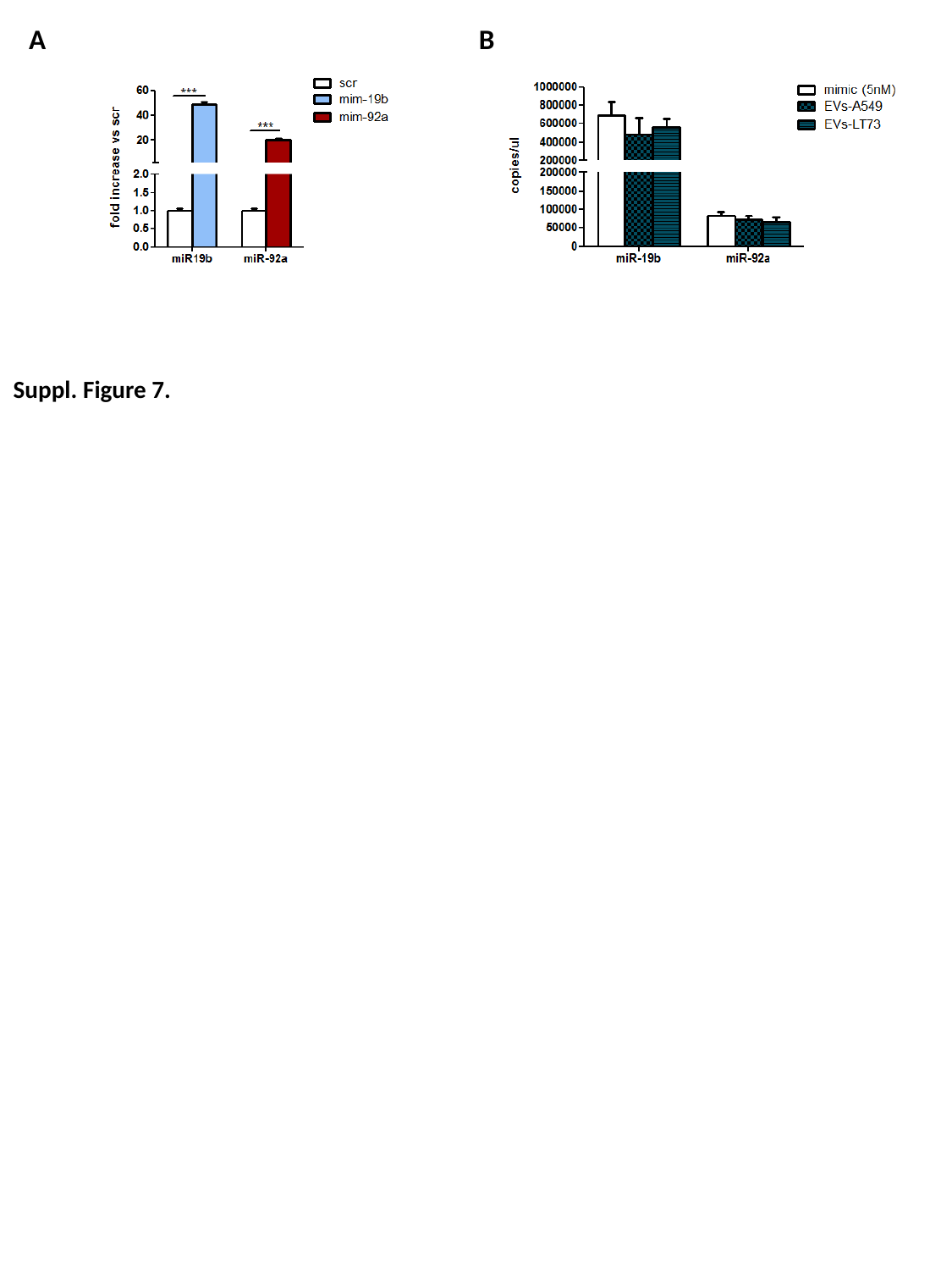

A
B
Suppl. Figure 7.

## Slide 8
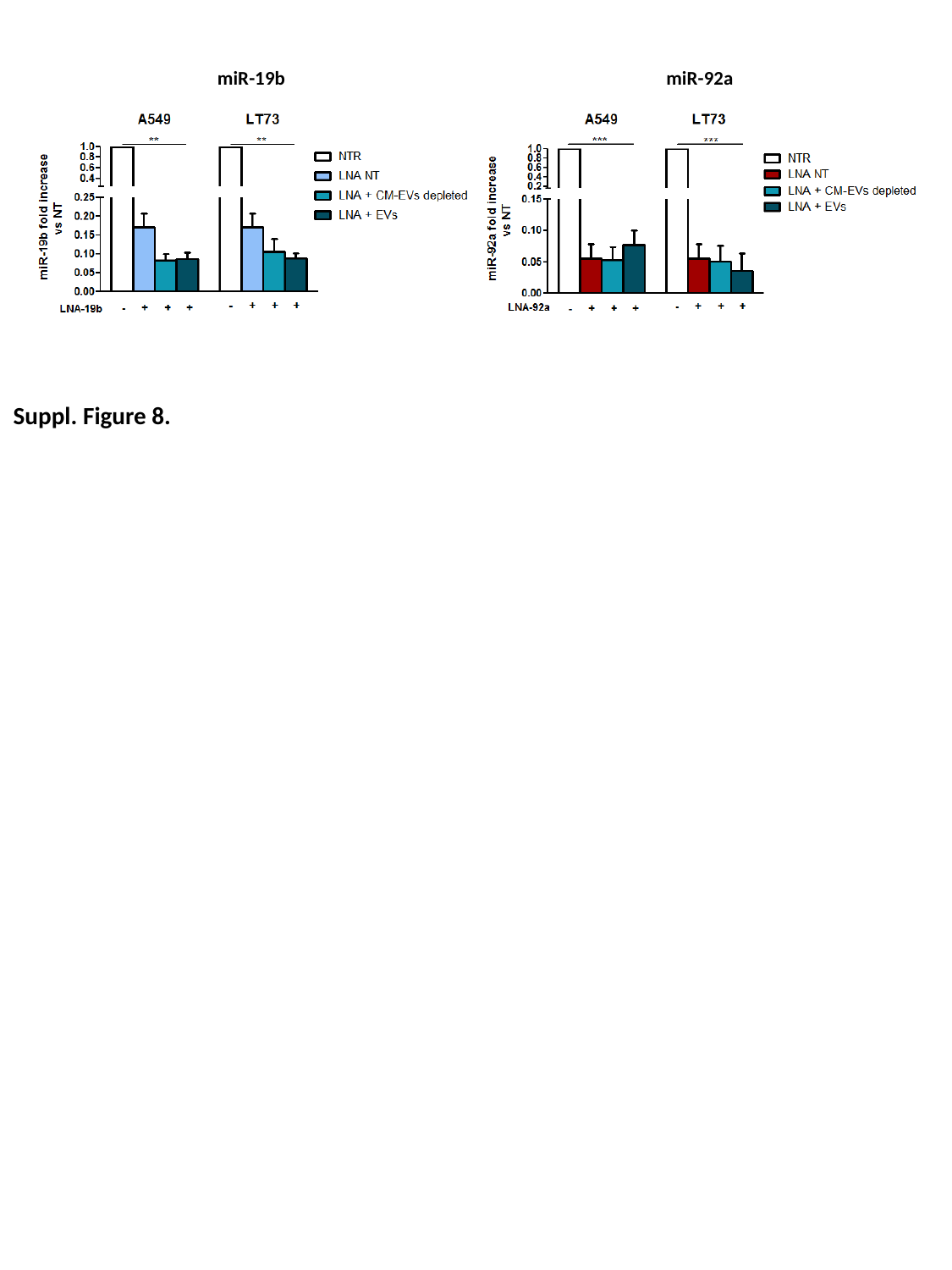

miR-19b
miR-92a
Suppl. Figure 8.

## Slide 9
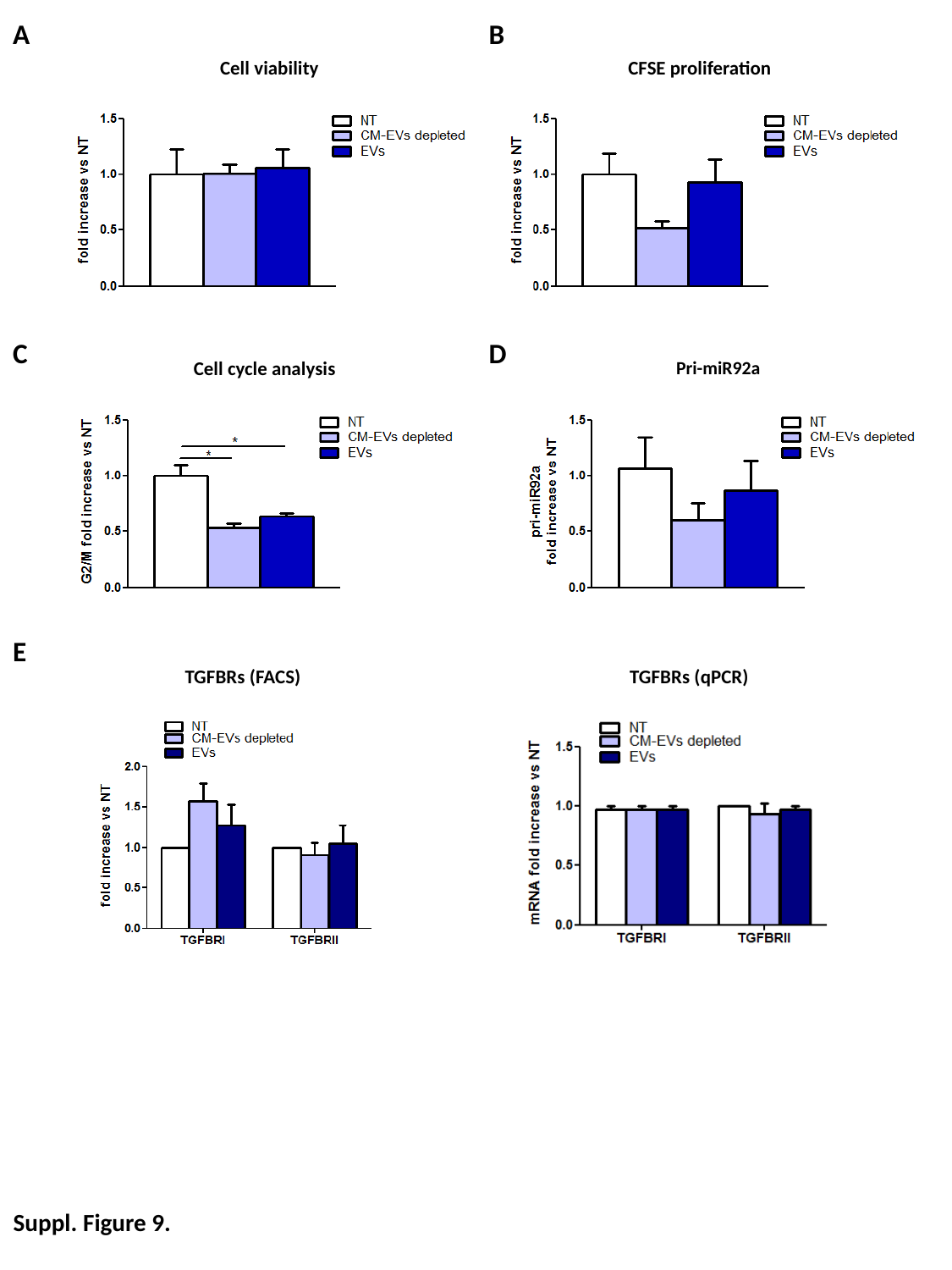

A
B
Cell viability
CFSE proliferation
C
D
Cell cycle analysis
Pri-miR92a
E
TGFBRs (FACS)
TGFBRs (qPCR)
Suppl. Figure 9.

## Slide 10
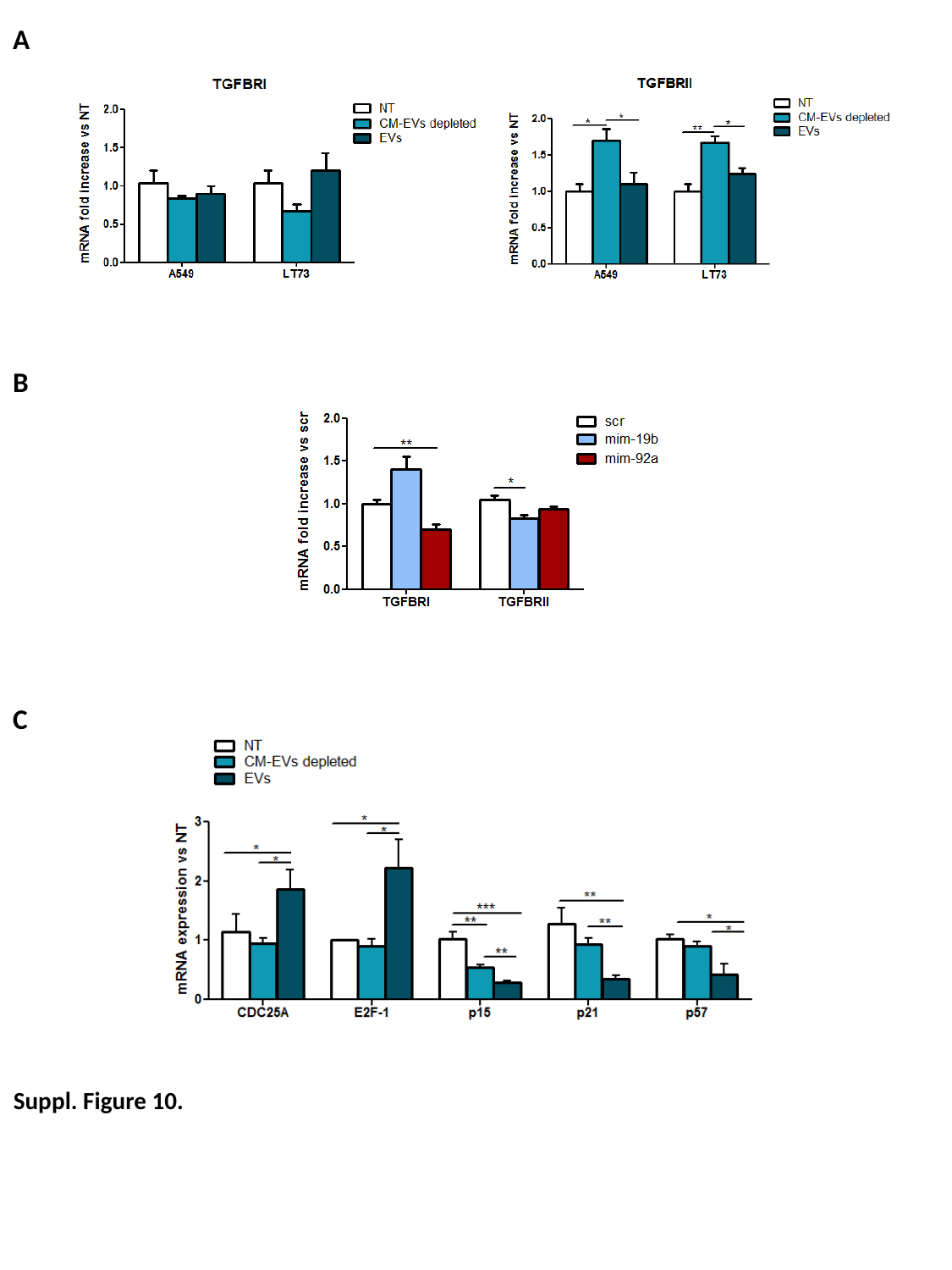

A
B
C
Suppl. Figure 10.

## Slide 11
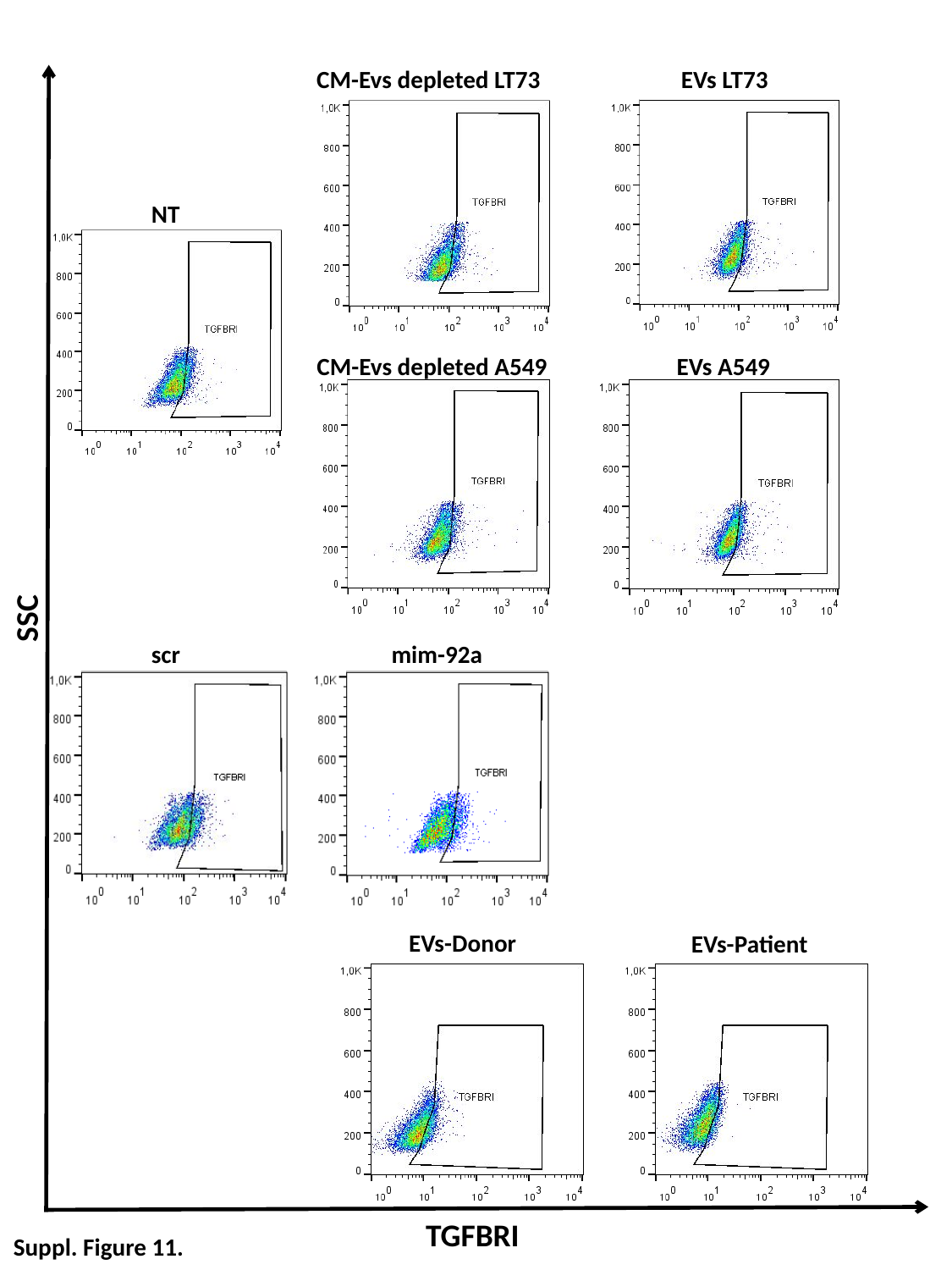

CM-Evs depleted LT73
EVs LT73
NT
CM-Evs depleted A549
EVs A549
SSC
scr
mim-92a
EVs-Donor
EVs-Patient
TGFBRI
Suppl. Figure 11.

## Slide 12
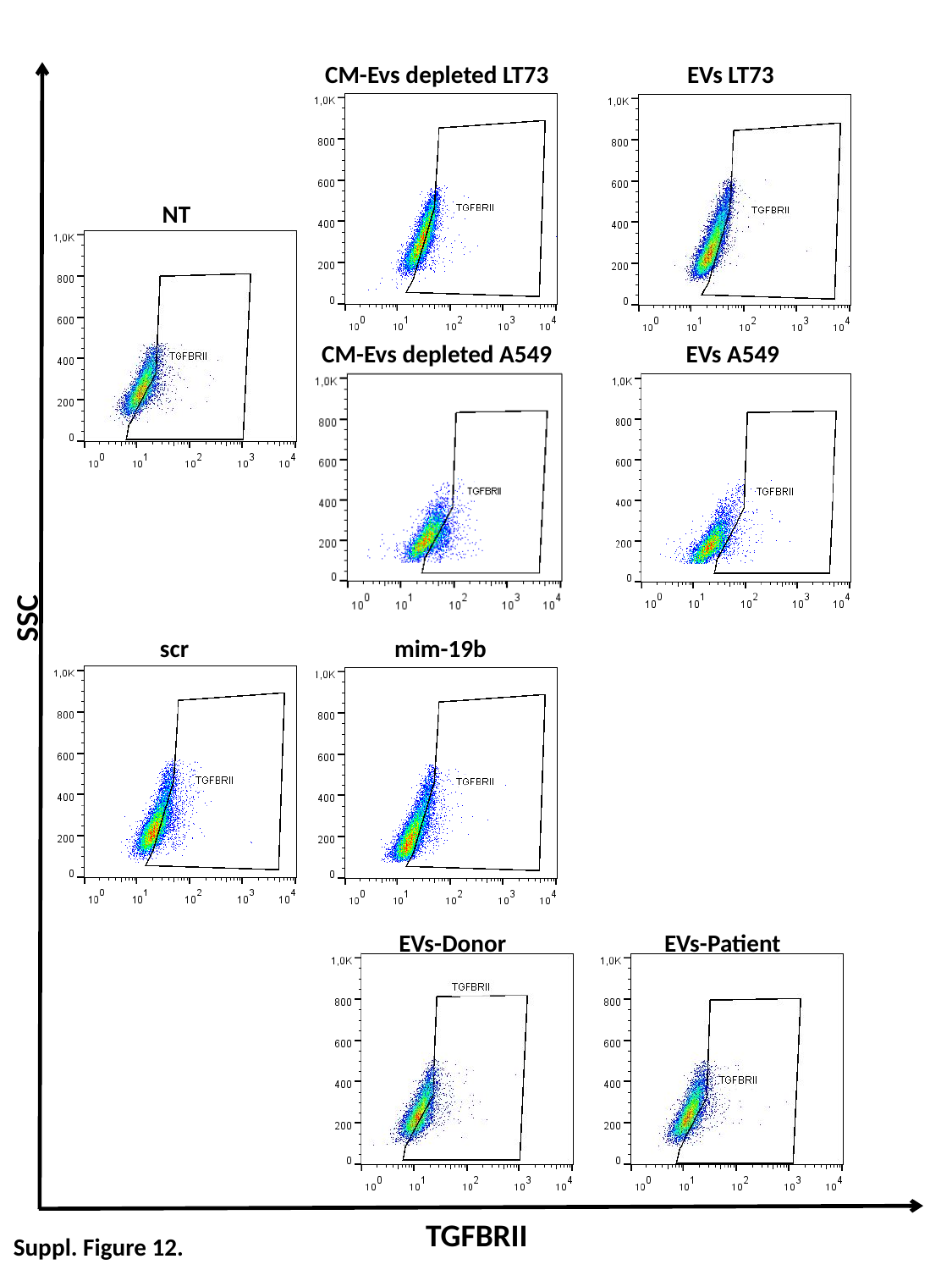

CM-Evs depleted LT73
EVs LT73
NT
CM-Evs depleted A549
EVs A549
SSC
scr
mim-19b
EVs-Donor
EVs-Patient
TGFBRII
Suppl. Figure 12.

## Slide 13
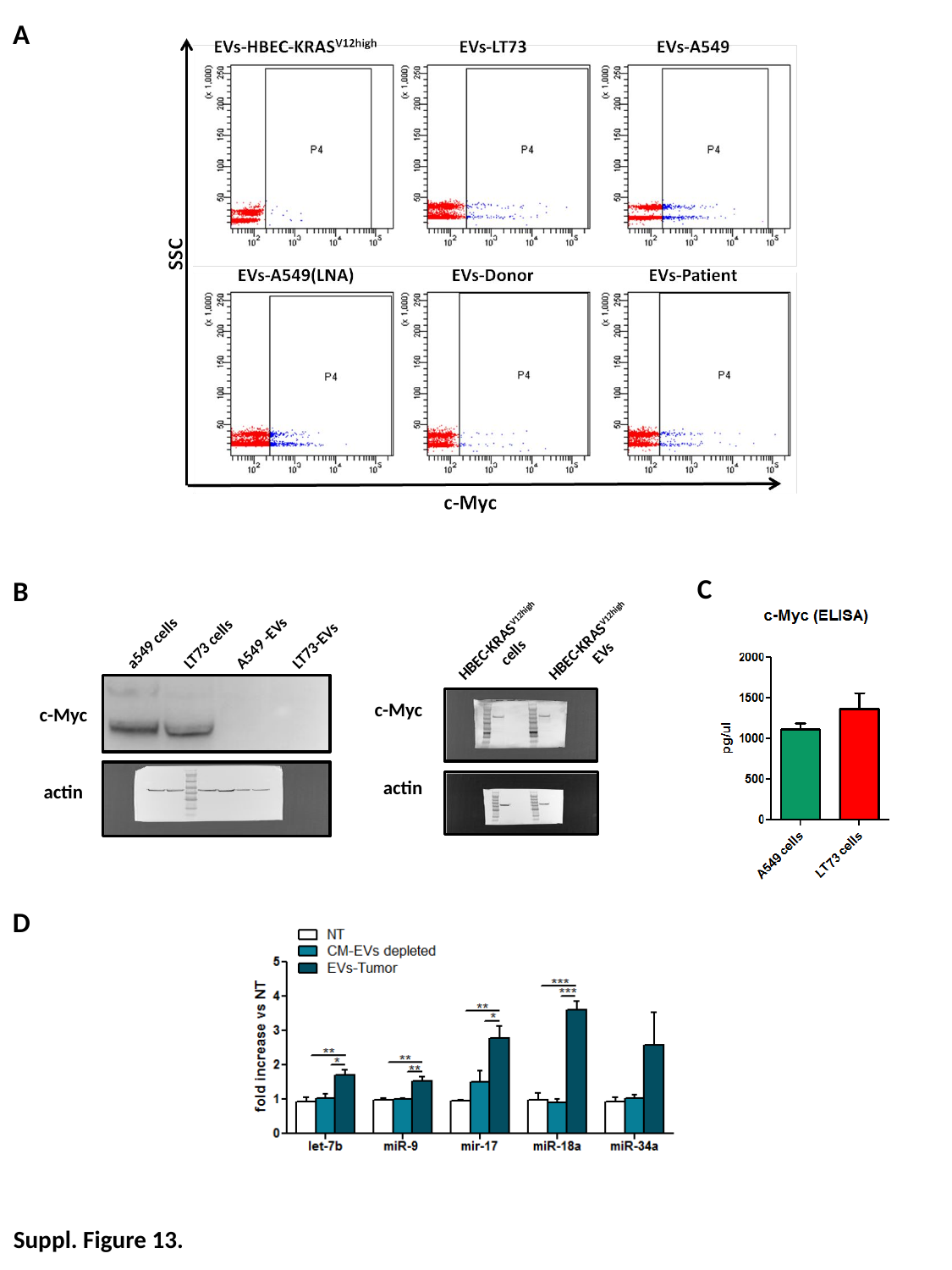

A
C
B
a549 cells
LT73 cells
A549 -EVs
LT73-EVs
c-Myc
actin
HBEC-KRASV12high
cells
HBEC-KRASV12high
EVs
c-Myc
actin
D
Suppl. Figure 13.

## Slide 14
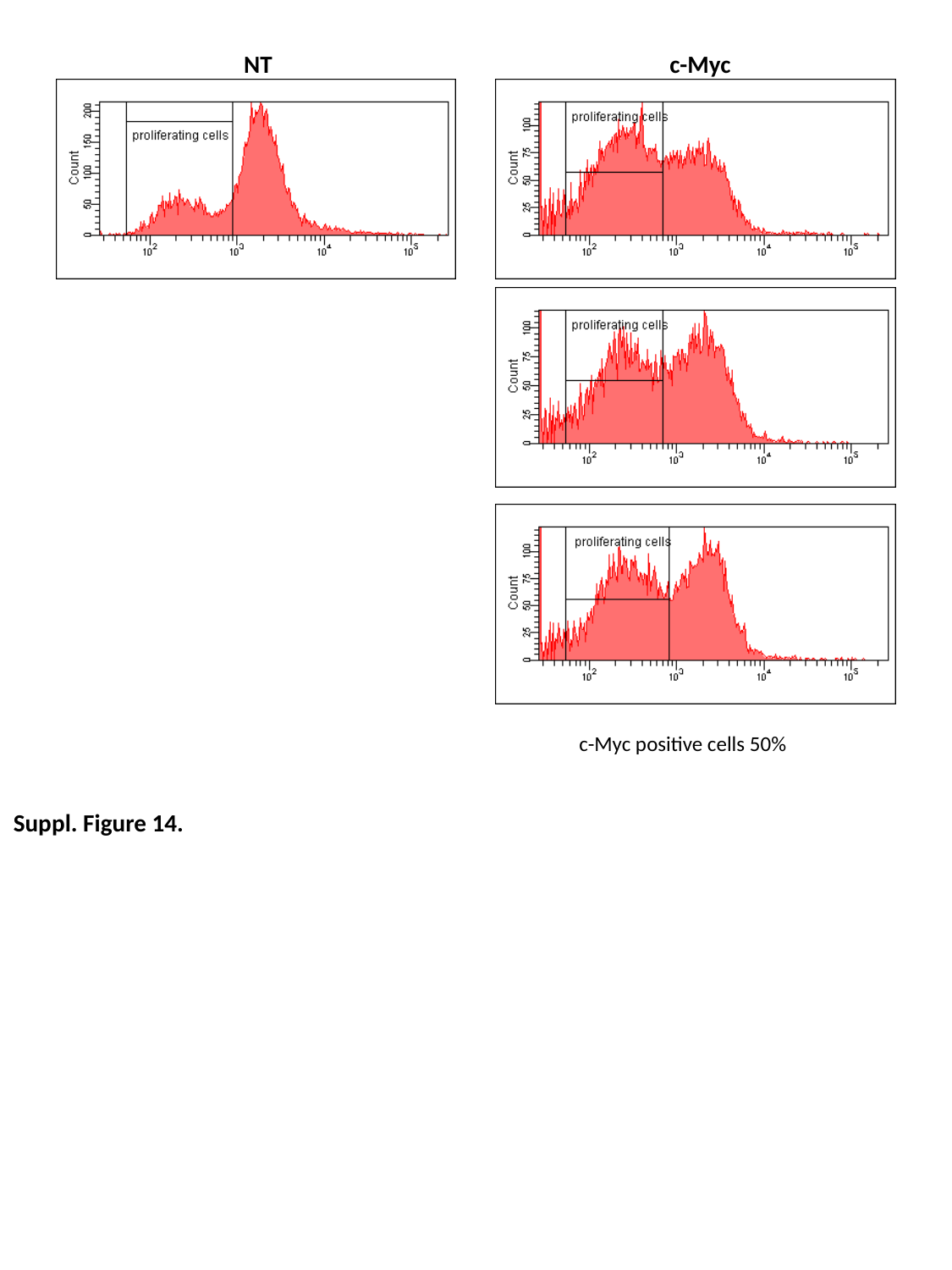

NT
c-Myc
c-Myc positive cells 50%
Suppl. Figure 14.

## Slide 15
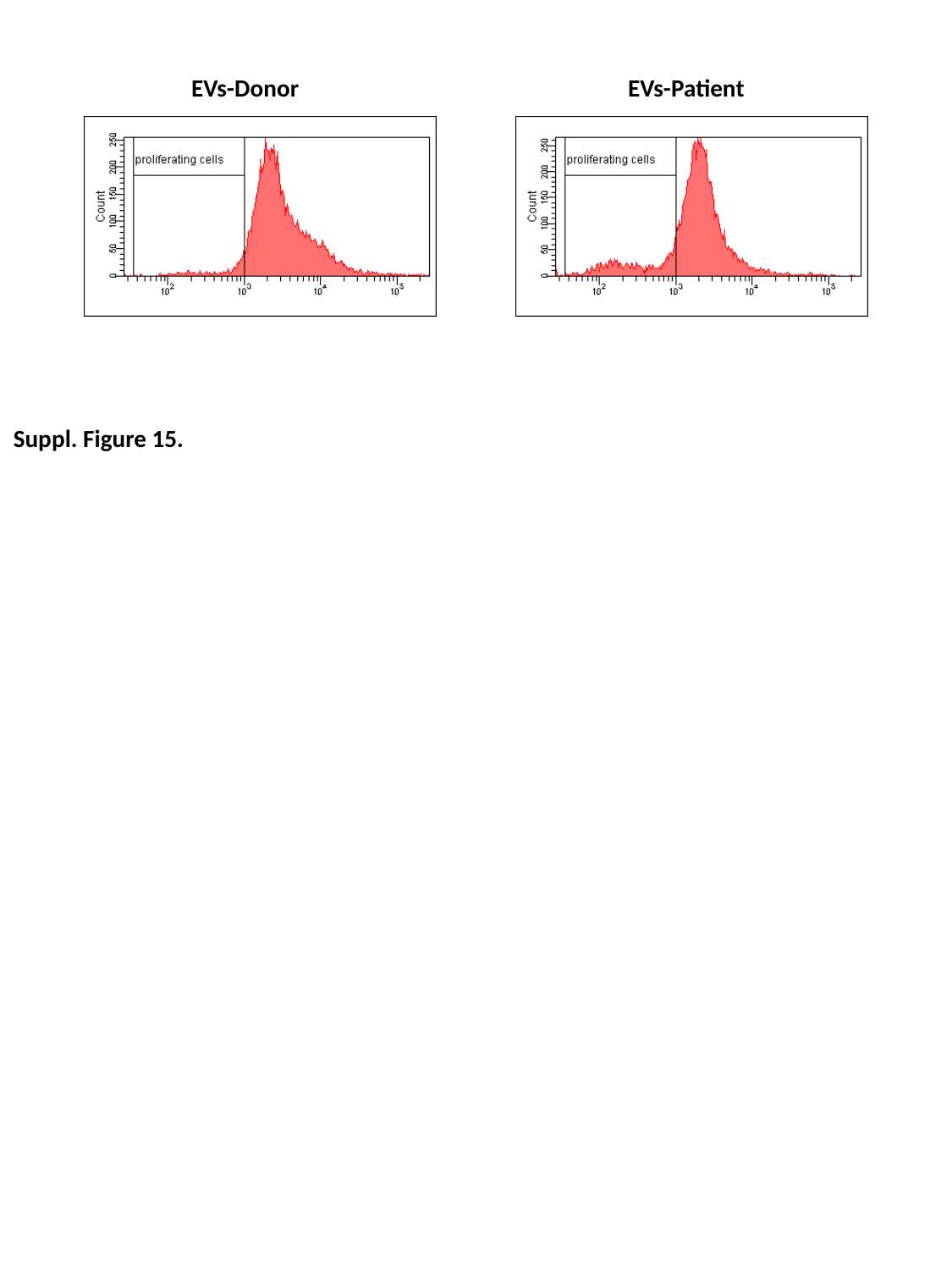

EVs-Donor
EVs-Patient
Suppl. Figure 15.

## Slide 16
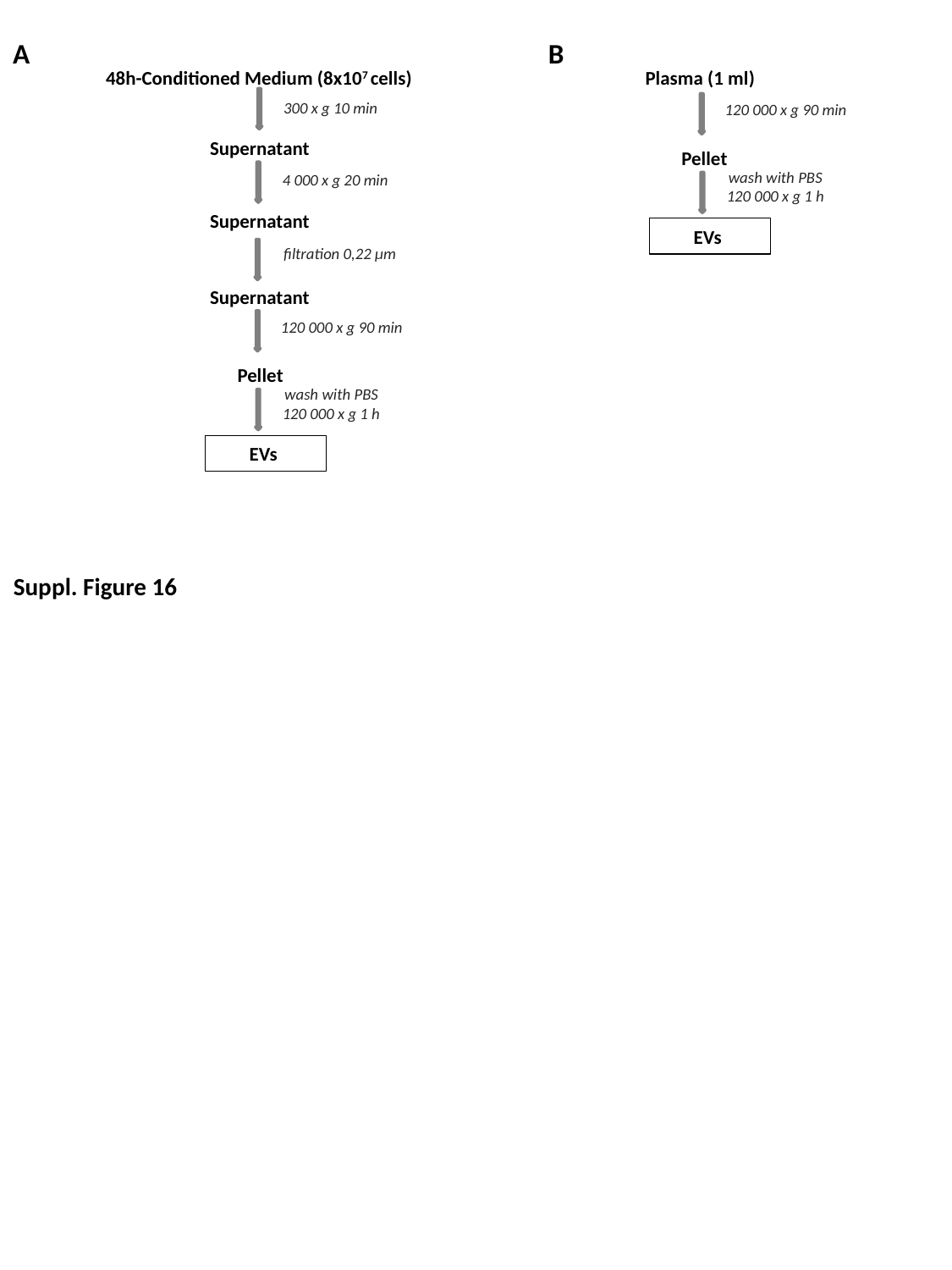

A
B
48h-Conditioned Medium (8x10^7^ cells)
300 x g 10 min
Supernatant
4 000 x g 20 min
Supernatant
filtration 0,22 µm
Supernatant
120 000 x g 90 min
Pellet
wash with PBS
120 000 x g 1 h
EVs
Plasma (1 ml)
120 000 x g 90 min
Pellet
wash with PBS
120 000 x g 1 h
EVs
Suppl. Figure 16

## Slide 17
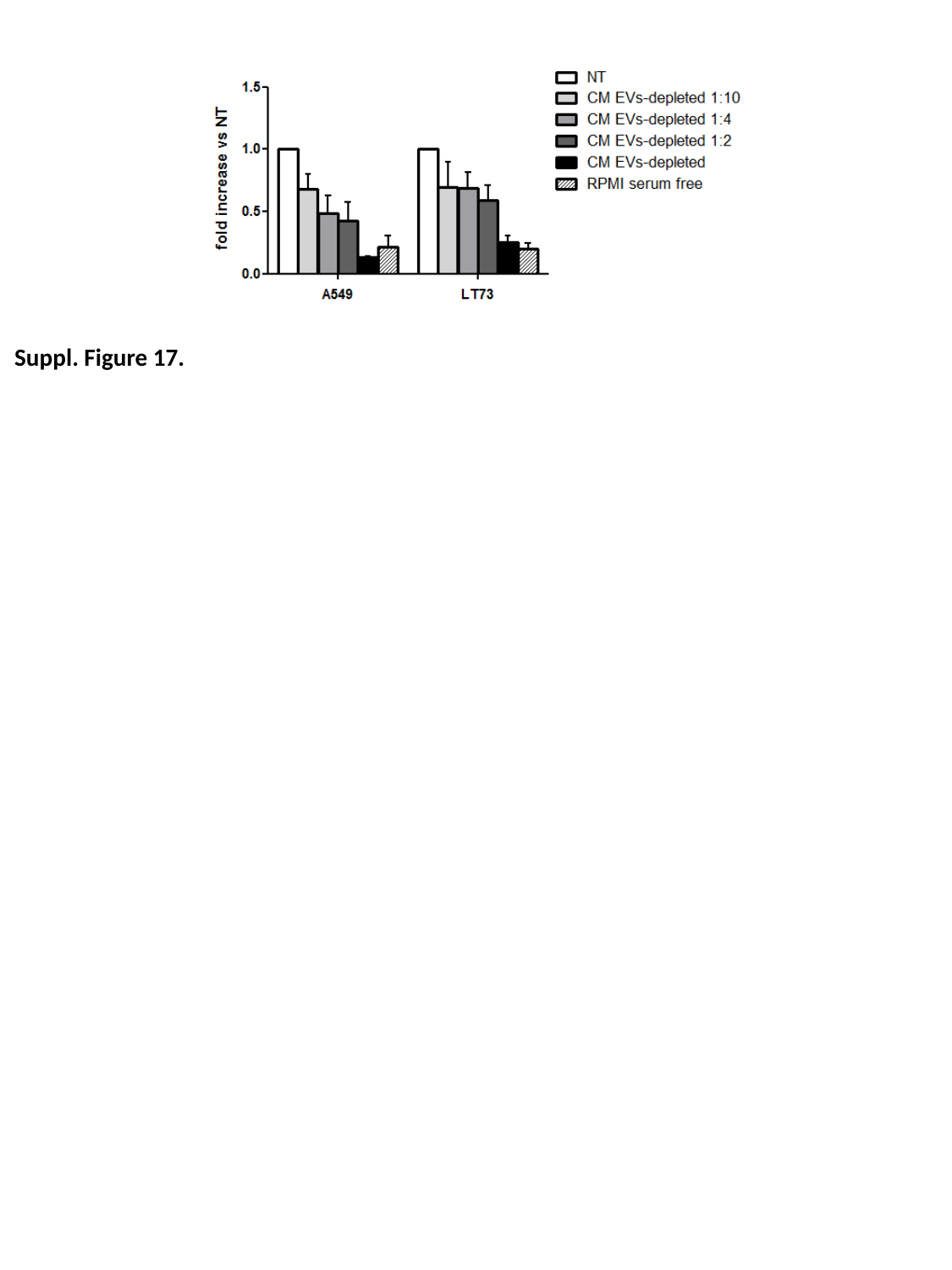

Suppl. Figure 17.
